# Metabolomic and transcriptomic responses of Adiantum (*Adiantum nelumboides*) leaves under drought, half-waterlogging, and rewater conditions

**DOI:** 10.3389/fgene.2023.1113470

**Published:** 2023-04-17

**Authors:** Qianyan Liang, Bicheng Dun, Linbao Li, Xiaobo Ma, Haibo Zhang, Yang Su, Di Wu

**Affiliations:** ^1^ Rare Plants Research Institute of Yangtze River, Three Gorges Corporation, Yichang, Hubei Province, China; ^2^ National Engineering Research Center of Eco-Environment Protection for Yangtze River Economic Belt, Beijing, China

**Keywords:** water stress, flavonoid biosynthesis, photosynthesis, fatty acid, abscisic acid, ROS scavenging

## Abstract

**Introduction:** *Adiantum nelumboides* (Adiantum) is an endangered fern with a narrow distribution along the Yangtze River in China. Due to its cliff-dwelling habit, it experiences water stress conditions, which further endangers its survival. However, no information is available about its molecular responses to drought and half-waterlogging conditions.

**Methods:** Here, we applied five and ten days of half-waterlogging stress, five days of drought stress, and rewatering after five days of drought stress, and studied the resulting metabolome profiles and transcriptome signatures of Adiantum leaves.

**Results and Discussion:** The metabolome profiling detected 864 metabolites. The drought and half-waterlogging stress induced up-accumulation of primary and secondary metabolites including amino acids and derivatives, nucleotides and derivatives, flavonoids, alkaloids, and phenolic acid accumulation in Adiantum leaves. Whereas, rewatering the drought-stressed seedlings reversed most of these metabolic changes. Transcriptome sequencing confirmed the differential metabolite profiles, where the genes enriched in pathways associated with these metabolites showed similar expression patterns. Overall, the half-waterlogging stress for 10 days induced large-scale metabolic and transcriptomic changes compared to half-waterlogging stress for 05 days, drought stress for 05 days or rewatering for 05 days.

**Conclusion:** This pioneering attempt provides a detailed understanding of molecular responses of Adiantum leaves to drought and half-waterlogging stresses and rewater conditions. This study also provides useful clues for the genetic improvement of Adiantum for drought/half-waterlogging stress tolerance.

## 1 Introduction

The *Adiantum nelumboides* (Adiantum) is a single-leaf evergreen plant of the Adiantaceae family (Smith et al., 2006). It has been used in Traditional Chinese Medicine (TCM) for over a century (Pryer et al., 2016). However, it has a narrow distribution along the Yangtze River and is endemic to Shizhu County, Sichuan province, China. Due to over-collection by local people for its medicinal uses, and the destruction of its habitats due to the construction of the Three Gorges Dam, it is now a nationally protected and endangered species (Liao et al., 2008). It is important to mention that it has also been listed as a class-I protected fern in China (Kang et al., 2008). Due to its rarity and cliff-dwelling habit where it grows on steep slopped rocks with thin soil, it often experiences water stress conditions, i.e., some places remain damp with excess water and others may experience limited water availability. In this regard, limited evidence is available on the responses of Adiantum to different water regimes. A 2008 study revealed that dry matter allocation, leaf relative area ratio, leaf relative water content, stomatal conductance, and water use efficiency were significantly affected in Adiantum grown in different levels of soil moisture (80%, 60%, and 40% water holding capacity) (Liao et al., 2008). Another study indicated that specific leaf area, root/shoot ratio, root mass, total mass, and water use efficiency were affected by drought stress. Among the two compared species, *Adiantum nelumboides* was relatively less affected by the imposed drought compared to*Adiantum capillus-veneris* (Liao et al., 2013). Other ferns exhibit a similar response to drought, i.e., relative leaf water content, osmotic substance contents, secondary metabolite accumulation, and increased activities of the reactive oxygen species (ROS) scavenging enzymes (Wang Y. et al., 2019). However, there are no studies that explored the transcriptomic and metabolomic changes in Adiantum grown under half-waterlogging, drought, and rewatering conditions. Nevertheless, this knowledge is needed for developing new genotypes of this class-I protected species, which can survive well under such stress conditions.

Plants adapt sophisticated responses to maintain optimal growth under drought-stress conditions. These include an array of short and/or long-term cellular and molecular responses to prevent water loss and help plants acquire resistance. Key changes are associated with abscisic acid (ABA) driven stomatal closure (Takahashi et al., 2020). Reports on lycophytes and ferns have suggested that stomatal closure is ABA-independent (Brodribb and McAdam, 2011). The sensing of drought and the production of responses involve hydraulic signals, changes in Ca^2+^ concentrations, production of ROS, phytohormone signals, and ion balance (Takahashi et al., 2020). The perception of drought stress is initiated in roots, which is then integrated into the distant organs, i.e., stem to leaves. Recently, a HYDROGEN-PEROXIDE-INDUCED Ca^2+^ INCREASE (HPCA) that sense increased ROS, e.g., H_2_O_2_ in guard cells, has been discovered (Wu et al., 2020). Changes in photosynthesis and transpiration due to drought stress (coupled with excess light) also disturb ROS/Ca^2+^ concentrations, which affect stomatal closure by changing gene expression related to guard cells (Kollist et al., 2019). Histidine kinases (HKs) also function as osmotic stress sensors (Sussmilch et al., 2017). However, the roles of Ca^2+^ channels as osmo-sensors is increasingly been explored (Yamanaka et al., 2010). Apart from these long-distance signals, some local signals such as changes in the expressions of genes associated with guard-cells i.e., CLAVATA3/EMBRYO-SURROUNDING REGION-RELATED (CLE) (Zhang et al., 2019), mitogen-activated protein kinases (MAPKs), and SLOW ANION CHANNEL ASSOCIATED 1 (SLAC1) mediate drought stress responses and offer stomatal control. Thus, ROS, turgor pressure, and Ca^2+^ waves regulate water loss in leaves under drought stress (Yuan et al., 2014). Nevertheless, the mechanisms of drought stress responses in plants are complex and their exploration continues.

Drought stress also triggers significant changes in the metabolic profiles of plants and their organs. The quantity and class of the accumulated compounds can be regarded as the function of plant responses and/or tolerance (Fàbregas and Fernie, 2019). Studies on different plant species have shown that soluble sugars, proline, amino acids and derivatives, flavonoids, and terpenoids are accumulated in drought-stressed plants (Du et al., 2012; Guo et al., 2018). The stress-induced accumulation of these metabolites enables the plants to maintain their growth by stabilizing protein structures, acting as free radical scavengers, and adjusting the redox balance (Yang et al., 2021). With the developments in transcriptome sequencing and metabolome profiling, it is now possible to collectively understand the expression changes and accumulation trends of genes and metabolites in species of interest under any stress (Hong et al., 2020; Wang et al., 2021). Considering the importance of the Adiantum in TCM, its narrow distribution, and its status as endangered species, we explored the leaf metabolome and transcriptome profiles under half-waterlogging, drought, and rewatered conditions. We present and discuss how these stresses affect the physiological and biochemical indicators in Adiantum. Such information is highly useful for the development of Adiantum genotypes with better adaptability to a broader distribution area under different water stress regimes.

## 2 Materials and methods

### 2.1 Plant material and stress treatments

*Adiantum nelumboides* X. C. Zhang (Adiantum) mother plant was collected from the Yangtze River Institute of Rare Plants, Hubei Province, China, and propagated by ramets to get individual plants in March 2021. A healthy and diseases free mother plant was selected. For this, robustly growing plants with normal leaf color, and without disease spots were selected. The unfolded first leaves were used for propagation. The daughter ramets were obtained by separating the soil blocks such that each block contained several daughter ramets. The soil blocks with daughter ramets were transferred to plastic pots (130 cm diameter) cleaned with 500 times chlorothalonil for 24 h and filled with peat, perlite, and river sand (3:1:1). The medium was sterilized by spraying with potassium permanganate 2000 times solution and stirred evenly. Before its transfer to plastic pots, the medium was sealed with a plastic film and left in Sun for 2 days. The daughter ramets were then allowed to grow till 3-4 leaf stage. Nine healthy and uniform plants were selected for each treatment, i.e., there were three replicates with three plants each.

A pilot experiment was conducted to determine the number of days required for each stress treatment. For drought stress treatment, After 5 days of drought (SW05), the leaves appeared curly, thus it was selected for further analyses. For the drought stress treatment, the water of the selected pots was withheld and when the pot soil water content remained 30% (w/w) it was considered as the start of the drought stress, which was then continued for 5 days until sampled. After 5 days of drought, the rewatering treatment was carried out. For rewatering, the selected drought stress plants were watered to normal field capacity for 5 days (SWF05) and the samples were taken. The pilot experiment showed that the leaves were curled after half-waterlogging for 10 days and the survival rate was affected. For half-waterlogging stress, the pots were irrigated till the water level was above the surface of basin soil. The half-waterlogging treatment was carried out for five (DW05) and 10 days (DW10).

### 2.2 Preparation of samples and extraction for metabolome assays

Freeze-dried triplicate biological samples (vacuum freeze-dryer, Scientz-100F) of SW05, SWF05, DW05, DW10, and CK were crushed using a mixer-mill (MM 400, Retsch). It involved zirconia beads and was processed at 30 Hz for 90 s. The lyophilized powder (100 mg) was dissolved in 1.2 mL methanol (70%) and vortexed six times for 30 s at an interval of 30 min. In the end, the samples were stored overnight at 4°C. The next day, the samples were centrifuged for 10 min at 12,000 rpm followed by a filtration using SCAA-104 with a pore size of 0.22 μm (ANPEL, Shanghai, China, http://www.anpel.com.cn/). The filtrates were then used for UPLC-MS/MS analysis.

### 2.2 UPLC conditions

A UPLC-ESI-MS/MS system (UPLC, SHIMADZU Nexera X2, https://www.shimadzu.com.cn/; MS, Applied Biosystems 4500 Q TRAP, https://www.thermofisher.cn/cn/zh/home/brands/applied-biosystems.html) was employed to analyze the sample extracts. Standard analytical settings were used as described below. UPLC: column, 1.8 µm, 2.1 mm * 100 mm (Agilent SB-C18); 0.1% formic acid in pure water represented the mobile phase, and acetonitrile with 0.1% formic acid represented the solvent B. A gradient program was used to perform measurements of samples. This program used A (95%) and B (5%) as starting conditions. During a period of 9 min, a linear gradient was established to A (5%) and B (95%). The terminal combination of A (5%) and B (95%) was sustained for 60 s. Afterward, within 1.1 min, the contribution of A and B was set to 95% and 5.0%, and it was sustained for 2.9 min. The flow velocity was adjusted to 0.35 mL per min. A 40°C temperature was used in the column oven. The volume of injection was 4 μL. An alternative connection of effluent was established to a QTRAP (ESI-triple quadrupole-linear ion trap)-MS.

### 2.3 ESI-Q TRAP-MS/MS

A triple quadrupole-linear ion trap mass spectrometer (Q TRAP; AB4500 Q TRAP UPLC/MS/MS System) was used to acquire the LIT and triple quadrupole (QQQ) scans. This system is equipped with an ESI Turbo Ion-Spray interface and it operates both the negative and positive ion modes. The Analyst 1.6.3 software (AB Sciex) is used to control the system’s functioning. The source operation parameters for ESI were designed as follows: turbo spray ion source; 550°C source temperature; the ion spray voltage was 5500 V for the positive ion mode) or −4500 V; the CUR (curtain gas), GSI (ion source gas I), GSII (gas II), were fixed at 25, 50, and 60 psi, respectively. The CAD (collision-activated dissociation) was set at high. A volume of 10 μmol/L polypropylene glycol solution was used for instrument tuning in QQQ and 100 μmol/L polypropylene glycol solution was used for mass calibration in LIT mode. The collision gas (nitrogen) was set to medium during QQQ scans in MRM experiments. DP and CE for individual MRM transitions were done with further DP and CE optimization. A specific set of MRM transitions were monitored for each period according to the metabolites eluted within this period.

### 2.4 Analytical methods

The statistics function “prcomp” within R (www.r-project.org) was used to perform the principal component analysis (unsupervised principal component analysis, PCA). Before unsupervised PCA, a unit variance scaling was executed on data. Heatmaps were used to represent the results of relative metabolites’ intensities. The “cor” function in R was used to calculate the Pearson’s correlation coefficients (PCC) between samples and visualized as heatmaps using the R package “ComplexHeatmap”. Unit variance scaled normalized signal intensities of metabolites in heatmap were pictured as a colored spectrum. A scale (VIP ≥1 and absolute log 2 FC (fold change) ≥ 1) was defined to identify significantly differentially accumulated metabolites between groups. The R package “MetaboAnalystR” was used to generate the OPLS-DA, score plots, and permutation plots. The OPLS-DA results were used to calculate the VIP values. However, before OPLS-DA, a log transformation (log 2) and mean centering was applied to the data. Moreover, a 200 permutations-based permutation test was also performed to avoid overfitting. The KEGG compound database (http://www.kegg.jp/kegg/compound/) was employed to annotate the identified metabolites following mapping through the KEGG pathway database (http://www.kegg.jp/kegg/pathway.html). Consequently, pathways mapped with significantly accumulated metabolites were used in metabolite sets enrichment analysis (MSEA). The *p*-values in hypergeometric test were used to determine their significance in MSEA.

### 2.5 Transcriptomic assay methods

The cDNA libraries were sequenced on the Illumina sequencing platform by Norminkoda Biotechnology Co., Ltd. (Wuhan, China). One percent agarose gel was used to monitor contamination and degradation of RNA. RNA purity and concentration were checked using the Nanodrop spectrophotometer (OD 260/280), whereas the Agilent 2100 detected the RNA integrity. After the quality testing, the library construction was carried out using following procedure: magnetic beads with Oligo (dT) were used to enrich eukaryotic mRNA and fragmentation buffer added to randomly interrupt mRNA. Afterward, this mRNA was used as a template to synthesize the cDNA strand using random primers and AMPure XP beads were used to purify cDNA followed by cDNA library synthesis using PCR enrichment. Finally, the quality of library was evaluated using Agilent Bioanalyzer 2100 system.

Cluster Generation System (cBot) was used through TruSeq PE Cluster Kit v3-cBot-HS (Illumia) to cluster the index-coded samples. Afterward, the libraries were sequenced on an Illumina Hiseq platform. The “fastp v0.19.3” was used to filter the obtained reads. It essentially removes the reads containing adapters and it also removes low-quality reads (N content of reads exceeds 10% of the base number of the reads). Moreover, the reads with Q ≤ 10 (quality value) representing more than 50% of the entire read were also removed. The clean reads were spliced using Trinity (Grabherr et al., 2011) to obtain reference sequences for subsequent analyses. All subsequent analyses were based on clean reads. The HISAT v2.1.0 (spliced ​​read mapper for RNA-Seq) was used for index building and comparing clean reads to the reference genome. The differential expression between the two groups was performed using DESeq2 v1.22.1, and Benjamini and Hochberg method correction was applied to the *p*-values.

The log2 foldchange and corrected *p*-value were used as a threshold to detect differentially expressed genes (DEGs). The False Discovery Rate (FDR) was calculated through correction of *p*-value. The screening conditions for differential genes were log2 foldchange ≥2 and FDR <0.01. A total of seven databases, namely, NR, SwissProt, COG, KOG, Pfam, GO, and KEGG were used for the annotation of identified genes and pathway annotation.

## 3 Results

### 3.1 Global metabolome dynamics

**FIGURE 1:**
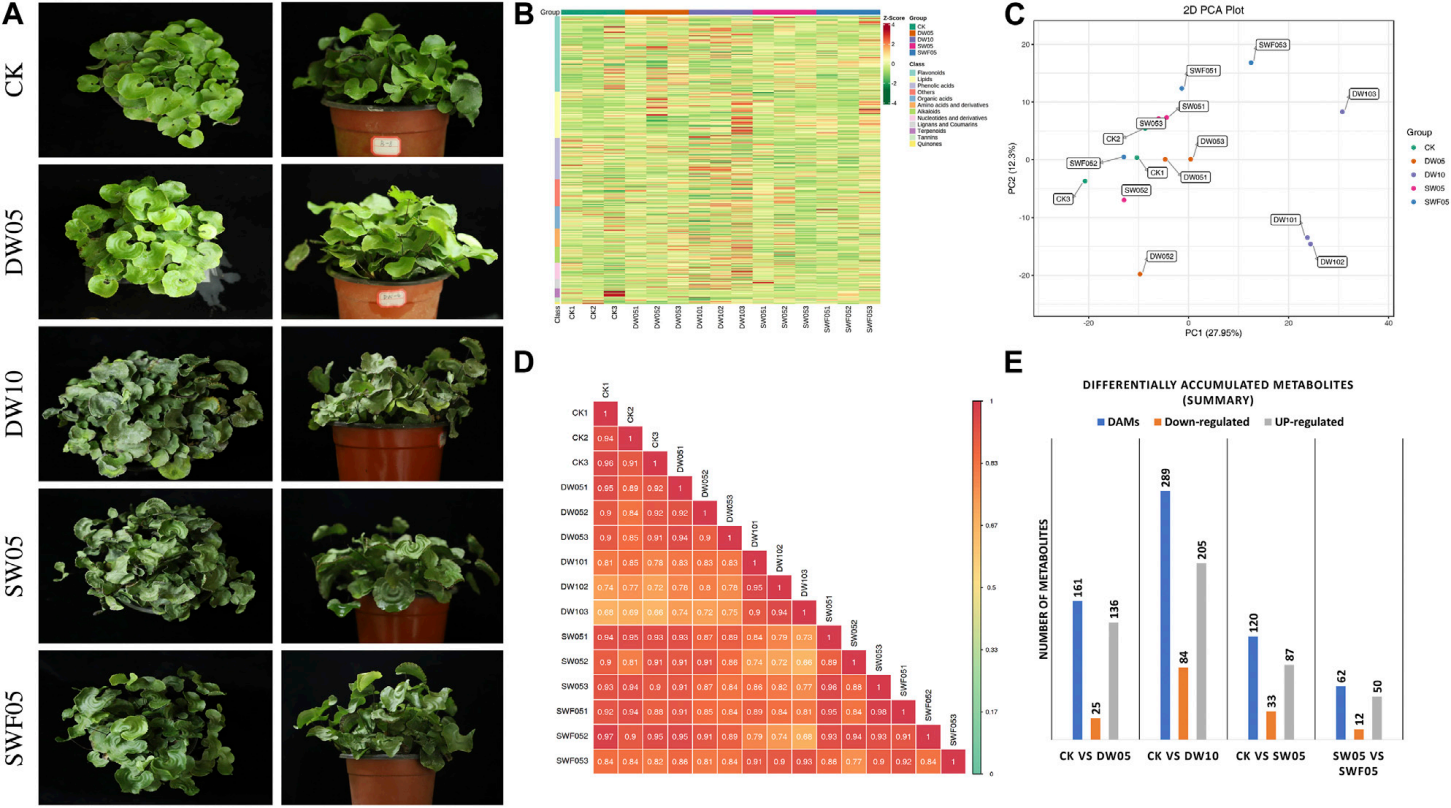
Overview of the observable phenotypes and summary of the metabolomic profiles in control and treatments. **(A)** Pictures of the Adiantum plants grown under different stress and control conditions. **(B)** Heatmap of the relative intensities of the metabolites accumulated in Adiantum leaves after different treatments. **(C)** Principal component analysis and **(D)** Heatmap of the Pearson’s Correlation Coefficient for the detected metabolites in Adiantum leaves after different stress treatments. **(E)** Summary of the differentially accumulated metabolites in Adiantum leaves under different stress conditions. Where, CK = control, DW05 = half-waterlogging for 5 days, DW10 = half-waterlogging for 10 days, SW05 = drought for 5 days, and SWF05 = day five after rewatering.

The Adiantum plants were grown under half-waterlogging (for five and 10 days), drought stress (for 5 days), and rewatering (for 5 days) and studied for their global metabolome profiles based on ultra-performance liquid chromatography/mass spectrometry (UPLC/MS) and gas chromatography/mass spectrometry (GC/MS) platform. The visible phenotypes showed that half-waterlogging for 10 days was more damaging to plants than 5 days. Similarly, drought also caused visible changes in the plant phenotype compared to CK, whereas rewatering reversed the drought effects (Figure 1A).

A total of 864 metabolites were detected based on the UPLC-MS/MS detection platform (Supplementary Table S1). These metabolites belong to 14 classes including alkaloids (48), amino acids and derivatives (55), flavonoids (227), lignans and coumarins (29), lipids (139), nucleotides and derivatives (48), organic acids (66), saccharides and alcohols (81), vitamins (17), others (07), phenolic acids (124), quinones (6), tannins (14), and terpenoids (27) (Figure 1B). The PCA of the metabolites detected in all stresses compared to CK showed that the treatment replicates tended to group together. The PC1 and PC2 explained 27.95% and 12.3% variations, respectively (Figure 1C). In case of the individual stress comparisons, i.e., CKvsDW05, CKvsDW10, CKvsSW05, and SW05vsSWF05, the % of the variations explained by PC1 and PC2 was higher (Supplementary Figure S1), indicating that these stresses affect the Adiantum metabolome. This was further supported by the fact that the PCC was 0.87 (Figure 1D). Overall, the number of differentially accumulated metabolites (DAMs) was highest for the samples treated with half-waterlogging for 10 days, i.e., DW10 (289 DAMs) followed by DW05 (161), SW05 (120), and samples harvested on the fifth day after rewatering (SW05 vs. SWF05; 62) (Figure 1E).

### 3.2 Differential metabolome profiles of Adiantum leaves subjected to drought stress, half-waterlogging stress, and rewatering

The screening criteria, i.e., metabolites with foldchange ≥2 and ≤0.5, and VIP ≥1 resulted in the identification of 161, 289, 120, and 62 DAMs between CKvsDW05, CKvsDW10, CKvsSW05, and SW05vsSWF05, respectively (Figure 1D). The half-waterlogging treatments (DW05 and DW10) induced significant increase in phenolic acids, vitamins, saccharides, alcohols, nucleotides and derivatives, lipids, lignans and coumarins, flavonoids, and amino acids and derivatives. Similarly, drought stress also induced an increased accumulation of these compound classes except terpenoids. The rewatered Adiantum leaves showed a decrease in both lipid and flavonoid content.

#### 3.2.1 Changes in the metabolome profile of Adiantum leaves in response to half-waterlogging stress

**FIGURE 2:**
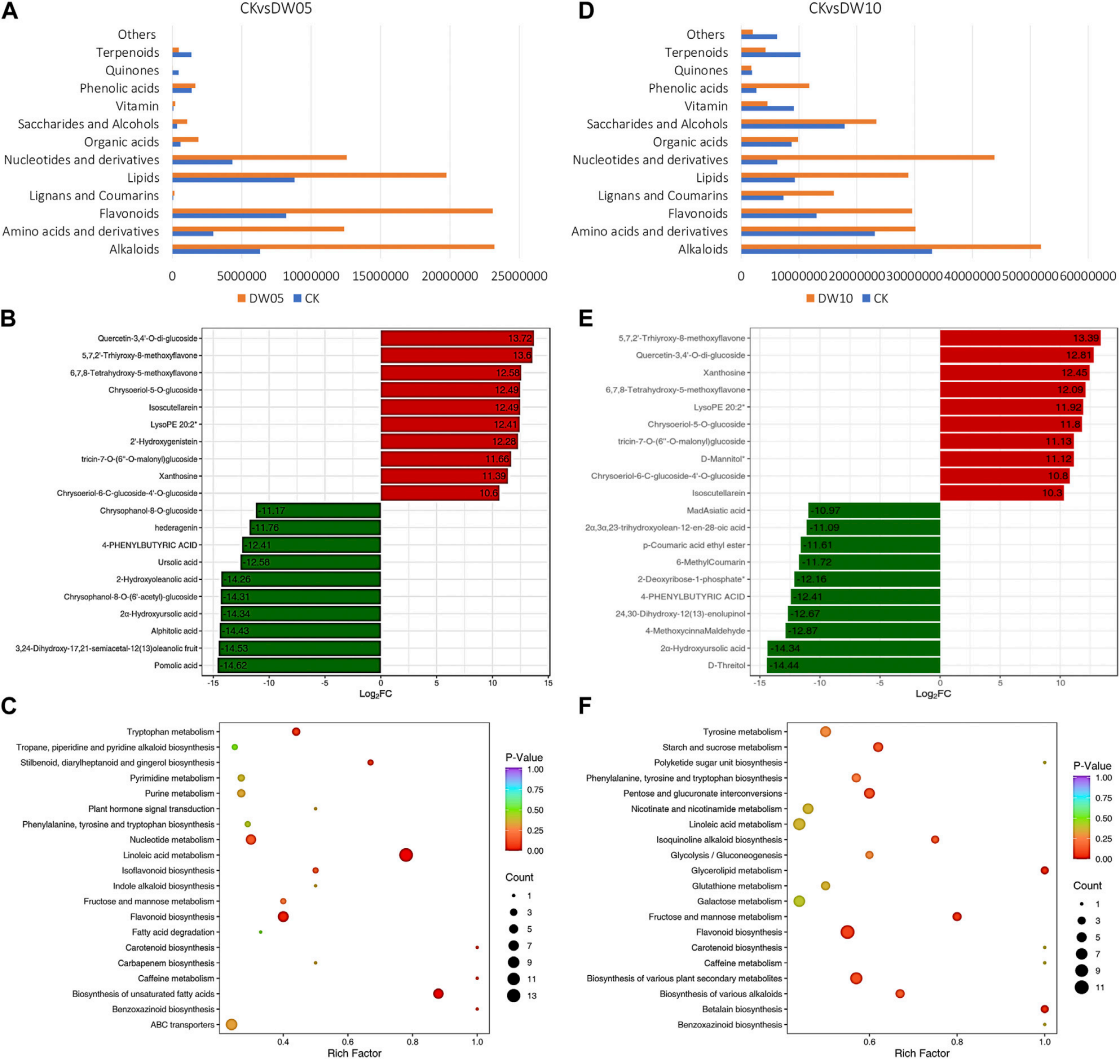
Half-waterlogging driven metabolomic changes in Adiantum leaves. **(A)** Changes in the accumulation pattern of major compound classes after 5 days of half waterlogging. The bars represent the sum of the relative intensities of compounds included in each class. **(B)** top and bottom 10 accumulated compounds in Adiantum leaves after 5 days of half waterlogging, and **(C)** scatter plot showing the pathways to which the DAMs were significantly enriched after 5 days of half waterlogging. **(D)** Changes in the accumulation pattern of major compound classes after 10 days of half waterlogging. The bars represent the sum of the relative intensities of compounds included in each class. **(E)** top and bottom 10 accumulated compounds in Adiantum leaves after 10 days of half waterlogging, and **(F)** scatter plot showing the pathways to which the DAMs were significantly enriched after 10 days of half waterlogging.

Adiantum leaves showed increased accumulation of most compound classes when exposed to half-waterlogging stress for 5 days (DW05). In total, we observed the differential accumulation of 161 metabolites (grouped in 11 compound classes) (Figure 1D; Figure 2A; Supplementary Table S1); where 25 and 136 metabolites were down- and up-accumulated, respectively, in DW05 compared to CK. Phenolic acids (7%), terpenoids (7%), nucleotides and derivatives (10%), lipids (23%), and flavonoids (29%) represented more than 70% of the DAMs. The top-five up-accumulated metabolites include isoscutellarein, chrysoeriol-5-O-glucoside, 6,7,8-tetrahydroxy-5-methoxyflavone, 5,7,2′-trhiyroxy-8-methoxyflavone, and quercetin-3,4′-O-di-glucoside. Similarly, top-five down-accumulated metabolites include chrysophanol-8-O-glucoside, 2α-hydroxyursolic acid, alphitolic acid, 3,24-dihydroxy-17,21-semiacetal-12(13)oleanolic fruit, and pomolic acid, (Figure 2B). These compounds were enriched in flavonoid biosynthesis, biosynthesis of unsaturated fatty acids, linoleic acid metabolism, nucleotide metabolism, stilbenoid, diarylheptanoid and gingerol biosynthesis, and tryptophan metabolism pathways (Figure 2C).

If half-waterlogging was prolonged to 10 days (DW10), the number of DAMs increased (289); 84 and 205 metabolites were down- and up-accumulated, respectively, in DW10 compared to CK. Organic acids (7%), saccharides and alcohols (9%), phenolic acids (14%), lipids (16%), and flavonoids (26%) represented more than 70% of the DAMs (Figure 2D). The top-five up-accumulated metabolites include lysoPE 20:2, 6,7,8-tetrahydroxy-5-methoxyflavone, xanthosine, quercetin-3,4′-O-di-glucoside, and 5,7,2′-trhiyroxy-8-methoxyflavone. Similarly, top-five down-accumulated metabolites (no flavonoids) include 4-phenylbutyric acid, 24,30-dihydroxy-12(13)-enolupinol, 4-methoxycinna maldehyde, 2α-hydroxyursolic acid, and D-threitol (Figure 2E). The DAMs in CKvsDW10 were significantly enriched in biosynthesis of alkaloids, secondary metabolite biosynthesis, flavonoid biosynthesis, fructose and mannose metabolism, glycerolipid metabolism, isoquinoline alkaloid biosynthesis, pentose and glucuronate interconversions, starch and sucrose metabolism, and tyrosine metabolism pathways (Figure 2F). These observations indicate that half-waterlogging causes a significant increase in secondary metabolites particularly flavonoids, terpenoids, and alkaloids. Moreover, if this condition is prolonged for 10 days, the sugar contents are also affected in the Adiantum leaves.

#### 3.2.2 Metabolomic response of Adiantum leaves to drought stress

**FIGURE 3:**
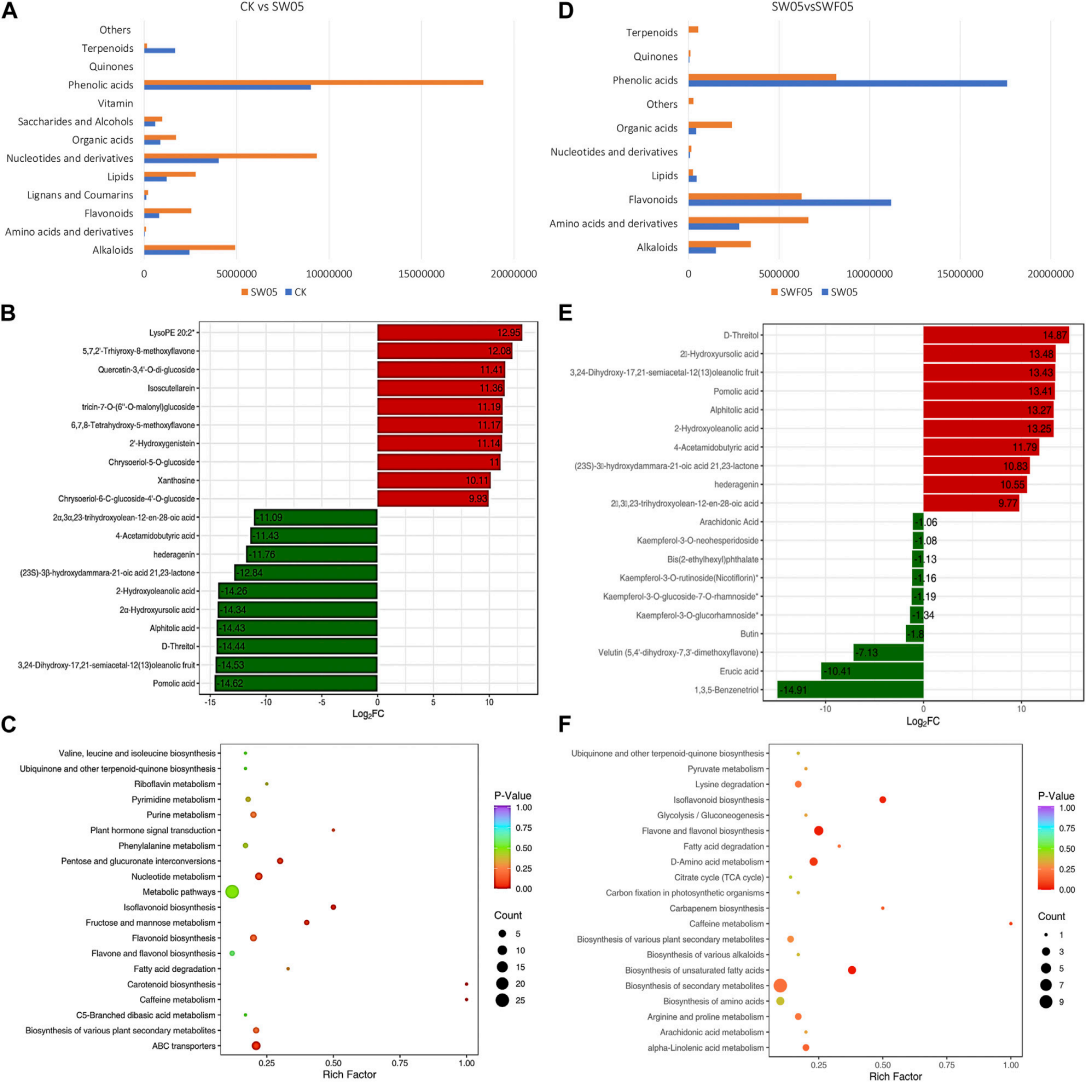
Drought stress and rewatering driven metabolomic changes in Adiantum leaves. **(A)** Changes in the accumulation pattern of major compound classes after 5 days of drought stress. The bars represent the sum of the relative intensities of compounds included in each class. **(B)** top and bottom 10 accumulated compounds in Adiantum leaves after 5 days of drought stress, and **(C)** scatter plot showing the pathways to which the DAMs were significantly enriched 5 days after drought stress. **(D)** Changes in the accumulation pattern of major compound classes on day five after rewatering. The bars represent the sum of the relative intensities of compounds included in each class. **(E)** top and bottom 10 accumulated compounds in Adiantum leaves on day five after rewatering, and **(F)** scatter plot showing the pathways to which the DAMs were significantly enriched on day five after rewatering.

The drought stress for 5 days resulted in the differential accumulation of 120 metabolites in Adiantum leaves. The most accumulated metabolites were phenolic acids, followed by nucleotides and derivatives, alkaloids, and flavonoids. Overall, we observed a reduction in terpenoid content, whereas other metabolite classes accumulated in significantly higher quantities after drought stress (Figure 3A). Drought resulted in the higher accumulation of 5,7,2′-trhiyroxy-8-methoxyflavone, quercetin, isoscutellarein, tricin-7-O)6″-O-malonyl)glucoside, and 2′-hydroxygenistein. Whereas, pomolic acid, D-threitol, 2α-hydroxyursolic acid,2-hydroxyloleanolic acid, and 4-acetamidobutryic acid content decreased in SW05 compared to CK (Figure 3B). The 120 DAMs were enriched in different KEGG pathways of which notable were ABC transporters, biosynthesis of secondary metabolites, flavonoid biosynthesis, fructose and mannose metabolism, isoflavonoid biosynthesis, pentose and glucuronate interconversions, and nucleotide metabolism (Figure 3C).

#### 3.2.3 Changes in the metabolome profile of the Adiantum leaves after rewatering

We also checked the changes in the metabolome profiles of the Adiantum leaves harvested on the fifth day after rewatering. For this, we rewatered the drought-stressed Adiantum plants and observed their metabolome profiles on day five of rewatering. Only 62 metabolites were differentially accumulated between SW05 and SWF05; 50 and 12 metabolites were up- and down-accumulated, respectively, in SWF05 compared to SW05. Alkaloids (8%), aminoacids & derivatives (15%), flavonoids (28%), and phenolic acids (41%) represented more than 90% of the DAMs. The rewatering induced upregulation of some saccharides and alcohols, terpenoids, phenolic acids, lipids, and flavonoids contents (Figure 3D). Clearly, the up-accumulation of a relatively higher number of metabolites indicates that the drought stress effects are being reverted in Adiantum leaves. The top-five down-accumulated metabolites in SWF05 compared to SW05 were 1,3,5-benzenetriol, erucic acid, velutin (5,4′-dihydroxy-7,3′-dimethoxyflavone), butin, and kaempferol-3-O-glucorhamnoside. Contrarily, the top-five up-accumulated metabolites in SWF05 compared to SW05 include alphitolic acid, pomolic acid, 3,24-dihydroxy-17,21-semiacetal-12(13)oleanolic fruit, 2α-hydroxyursolic acid, and D-threitol (Figure 3E). The DAMs in SW05vsSWF05 were enriched in biosynthesis of unsaturated fatty acids, isoflavonoid biosynthesis, alpha-linolenic acid metabolism, and biosynthesis of secondary metabolites pathways (Figure 3F).

### 3.3 Transcriptomic response of Adiantum leaves to drought, rewatering, and half-waterlogging stress

A total of 05 samples (15 libraries) were analyzed by transcriptome sequencing. The number of clean reads of each cDNA library was between 62 and 92 million reads; 109 Gb clean reads were obtained. The Q20% and Q30% were >97% and >93%, respectively, and the GC content was ∼49%. Of the 291,728 transcripts, 221,659 were assembled into unigenes and 36,591 coding genes (Supplementary Table S2). All the unigenes could be annotated in different databases; KEGG (33%), NR (83%), SwissProt (33%), eggNOG (53%), GO (59%), and Pfam (87%) (Supplementary Table S3).

**FIGURE 4:**
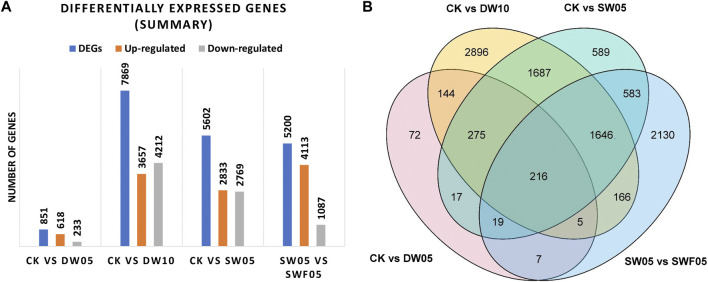
Summary of differential gene expression in Adiantum leaves after half-waterlogging, drought stress, and rewatering conditions. **(A)** Bar chart showing the number of differentially expressed genes and **(B)** Venn diagram of differential genes in Adiantum leaves. Where, CK = control, DW05 = half-waterlogging for 5 days, DW10 = half-waterlogging for 10 days, SW05 = drought stress for 5 days, and SWF05 = day five after rewatering.

The half-waterlogging for 10 days significantly affected the Adiantum leaves (Figure 4A) as evidenced by the highest number of DEGs in CKvsDW10 (7869), which is consistent with the metabolome profiles of the half-waterlogging (for 10 days) stressed Adiantum leaves (Figure 2D). Whereas, CKvsSW05, SW05vsSWF05, and CKvsDW05 had 5602, 5200, and 851 DEGs, respectively (Figure 4A). A total of 216 DAMs were common among all the treatments (Figure 4B). Moreover, 72, 2896, 589, and 2130 genes were uniquely regulated in CKvsDW05, CKvsDW10, CKvsSW05, and CKvsSWF05, respectively (Figure 4B; Supplementary Table S4).

#### 3.3.1 Transcriptomic sequencing confirms metabolomic changes in Adiantum leaves in response to half-waterlogging

**FIGURE 5:**
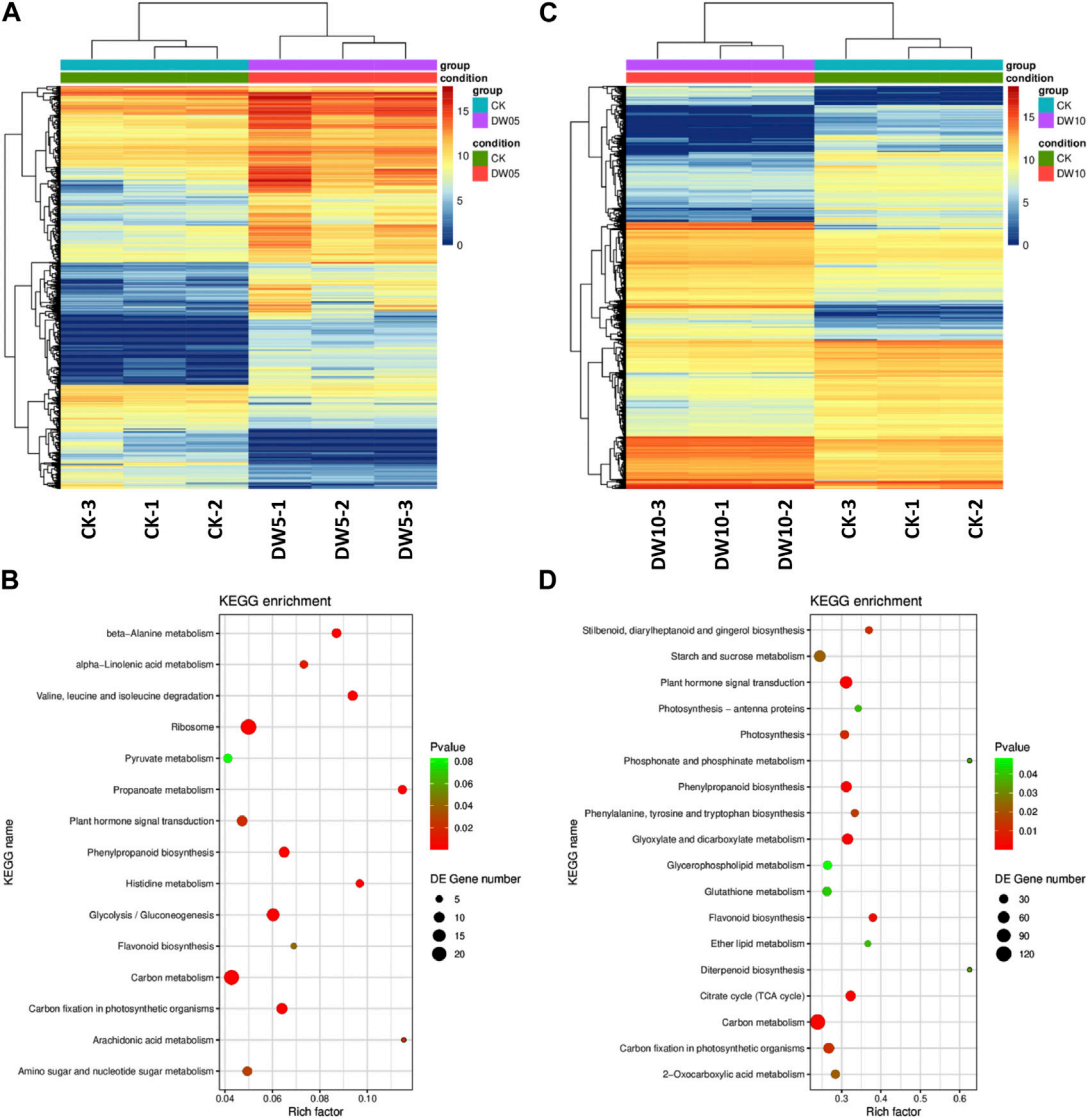
Half-waterlogging stress driven transcriptome changes in Adiantum leaves. **(A)** Heat map representation of expression profiles for differentially regulated genes in response to 5 days of half-waterlogging stress. **(B)** Heat map representation of expression profiles for differentially regulated genes in response to 10 days of half-waterlogging stress. **(C)** Scatter plot showing the pathways to which the DEGs were significantly enriched after 5 days of half-waterlogging. **(D)** Scatter plot showing the pathways to which the DEGs were significantly enriched after 10 days of half-waterlogging. Where, CK = control, DW05 = half-waterlogging for 5 days, and DW10 = half-waterlogging for 10 days.

Half-waterlogging caused a significant increase in the accumulation of lipids, phenolic acids, alcohols, flavonoids, amino acids and derivatives, and alkaloids (Figure 2). In this regard, we checked the changes in the expressions of the related genes. Like the higher number of compounds accumulated in response to DW10, we also observed that a relatively higher number of genes were expressed in DW10 compared to DW05 (Figure 4A; Figure 5). Of the 15 transcripts enriched in glycolysis, 11 were upregulated in DW05 compared to CK (Table 1; Supplementary Table S5). These upregulated genes included phosphate dehydrogenase (GAPDH), glyceraldehyde-3-phosphate dehydrogenase (ALDO), pyruvate decarboxylase (pdc), glyceraldehyde-3-phosphate dehydrogenase (GAPCP2), aldehyde dehydrogenase family 2 member B7 (ALDH2b), beta-enolase (ENO), and alcohol dehydrogenase 1 (ADH1). Interestingly, we observed that the expression values of these genes were higher in DW10 compared to CK as well as DW05, indicating that the increased duration of half-waterlogging further induces changes in glycolysis/gluconeogenesis. These observations are consistent with the changes in the expression of genes enriched in photosynthesis as well as other related pathways, i.e., antenna proteins and carbon fixation (Figure 5). Only one gene photosystem II CP43 chlorophyll apoprotein (psbC, g495_i0) was upregulated in DW05 compared to CK, whereas, 28 transcripts enriched in photosynthesis showed decreased expressions. Similarly, none of the antenna proteins showed changes in expressions in DW05, whereas thirteen transcripts annotated as chlorophyll a-b binding protein 5, Lhca4 protein, PSI antenna protein Lhca1, PSI antenna protein Lhca3, Chlorophyll A-B binding protein, chlorophyll a-b binding protein of LHCII type III, and protein lhcb4 were downregulated in DW10. These expression changes indicate that DW05 slightly affects photosynthesis and energy harvesting complexes, whereas, if half-waterlogging is prolonged to 10 days, it significantly affects the antenna proteins and photosynthesis. Possibly, this effect is further transferred down to other pathways, i.e., carbon fixation in photosynthetic organisms. In this pathway, 11 genes were differentially expressed in DW05 (three of which were downregulated), whereas 45 genes were differentially expressed in DW10 (29 of which were downregulated) (Table 1; Supplementary Table S5).

**TABLE 1:** Details of important differentially expressed genes from different pathways in response to half-waterlogging, drought, and rewatering.

Pathway name	Gene name	Gene ID	CK vs. SW05	Ck vs. DW05	CK vs. DW10	SW05 vs. SWF05
Cutin, suberine, and wax biosynthesis	*CYP86B1*	g12531_i0	0	0	5.42	0.00
	*PXG2*	g235_i0	2.23	0	2.6	−1.77
	*HHT1*	g7973_i0	−2.73	0	−3.32	1.41
	*HHT1*	g23250_i0	−4.26	0	0	4.45
	*HHT1*	g17903_i0	−7.87	0	−11.51	4.96
Photosynthesis	*atpF*	g25060_i0	−2.48	0	−3.22	2.83
	*atpF*	g34976_i0	−1.35	0	−2.01	1.69
	*petE*	g28593_i0	0	0	−2.42	2.08
	petE	g36193_i0	−2.28	0	−3.01	2.75
	*petH*	g41544_i0	−2.22	0	−2.98	2.42
	*petH*	g85933_i0	−2.44	0	−3.19	2.63
	*petH*	g29429_i0	0	0	0	9.55
	*psbC*	g495_i0	3.05	2.22	0	−1.99
	*PsbO*	g24057_i0	−2.16	0	−3.95	2.42
	*PsbO*	g32853_i0	0	0	0	10.54
	*psbP*	g35684_i0	0	0	−1.37	0.00
	*psbP*	g43822_i0	−2.53	0	−3.36	2.83
	*psbP*	g14951_i0	0	0	−1.06	0.00
	*psbW*	g32401_i0	−1.71	0	−2.33	2.19
	*psbR*	g61708_i0	−1.95	0	−2.41	2.14
	*psbR*	g69970_i0	0	0	0	6.15
Photosynthesis - antenna proteins	*PSI antenna protein Lhca1*	g1469_i0	0	0	−2.39	0.00
	*PSI antenna protein Lhca1*	g27283_i0	0	0	−2.57	1.89
	*Chlorophyll A-B binding protein*	g43609_i0	0	0	0	9.31
	*Chlorophyll A-B binding protein*	g15421_i0	0	0	−2.32	0.00
	*PSI antenna protein Lhca3*	g45882_i0	−2.26	0	−3.84	2.53
	*PSI antenna protein Lhca3*	g9992_i0	−1.95	0	−2.88	2.24
	*Lhca4 protein*	g31431_i0	0	0	−2.94	2.16
	*chlorophyll a-b binding protein 5*	g48997_i0	0	0	−1.52	0.00
	*chlorophyll a-b binding protein 5*	g50984_i0	0	0	−1.55	0.00
	*LHCII type III*	g23723_i0	−1.96	0	−3.08	2.04
	*LHCII type III*	g27121_i0	0	0	−2.32	0.00
	*protein lhcb4*	g25283_i0	0	0	−2.93	2.20
	*protein lhcb4*	g27255_i0	0	0	0	9.67
	*protein lhcb4*	g33650_i0	0	0	0	10.20
Terpenoid backbone biosynthesis	*GGPS*	g9415_i0	10.7	7.51	11.86	0.00
	*HMGCR*	g8606_i0	2.13	0	2.73	0.00
	*DHDDS*	g14312_i0	2.17	0	0	−1.37
Phenylpropanoid biosynthesis	*4CL*	g10471_i0	0	−4.82	0	0.00
	*4CL*	g362_i0	1.53	0	1.88	−1.30
	*CA4H*	g20352_i0	0	0	5.85	0.00
	*CA4H*	g21065_i0	3.53	0	2.43	−2.21
	*CA4H*	g22027_i0	0	0	1.69	0.00
	*CAD*	g25451_i0	−2.92	−2.64	−2.4	1.72
	*CCoAMT*	g33096_i0	3.05	0	3.49	0.00
	*COMT*	g6175_i0	4.88	3.97	4.6	0.00
	*HCT*	g24768_i0	7.93	8.8	10.61	0.00
	*HCT*	g14561_i0	4.98	4.05	4.88	−1.76
	*HCT*	g12450_i0	0	7.4	7.09	0.00
	*HCT*	g16541_i0	−5.26	0	−4.59	3.67
	*HCT*	g21913_i0	−1.56	0	−1.71	1.47
	*HCT*	g4757_i0	−1.79	0	−2.14	0.00
	*HCT*	g18055_i0	−3.5	0	0	3.92
	*HCT*	g23429_i0	1.6	0	0	0.00
	*PAL*	g31530_i0	0	2.34	3.87	0.00
	*PNPC*	g33460_i0	0	7.64	8.11	0.00
	*PRDX6*	g1714_i0	2.87	0	2.82	0.00
	*PRDX6*	g33223_i0	0	0	−1.17	0.00
	*PRDX6*	g47492_i0	2.73	0	2.65	0.00
	*PRDX6*	g5902_i0	2.62	0	2.46	−1.43
	*PRDX6*	g61093_i0	3.02	0	2.95	0.00
	*PRDX6*	g72332_i0	2.53	0	2.39	0.00
	*PTAL*	g14807_i0	4.95	0	0	−4.85
Flavonoid biosynthesis	*F3′5′H*	g14562_i0	5.41	4.98	5.87	0.00
	*CA4H*	g19622_i0	−4.16	0	−5.09	4.73
	*CA4H*	g20352_i0	0	0	5.85	0.00
	*CA4H*	g21065_i0	3.53	0	2.43	−2.21
	*CHS*	g16571_i0	0	0	1.75	0.00
	*CHS*	g1849_i0	1.57	0	2.48	0.00
	*CHS*	g39825_i0	0	0	1.74	0.00
	*HCT*	g24768_i0	7.93	8.8	10.61	0.00
	*HCT*	g14561_i0	4.98	4.05	4.88	−1.76
	*HCT*	g12450_i0	0	7.4	7.09	0.00
	*HCT*	g16541_i0	−5.26	0	−4.59	3.67
	*HCT*	g20519_i0	5.56	0	4.93	0.00
	*HCT*	g21913_i0	−1.56	0	−1.71	1.47
	*HCT*	g26680_i0	6.55	0	5.91	−3.28
	*HCT*	g4757_i0	−1.79	0	−2.14	0.00
	*HCT*	g5363_i0	−4.05	0	−5.08	2.58
	*HCT*	g704_i0	2.2	0	2.17	0.00
	*HCT*	g18055_i0	−3.5	0	0	3.92
Plant hormone (Auxin)	*AUX1*	g10087_i0	6.67	−3.31	0	0.00
	*TIR1*	g6627_i0	−3.17	0	−2.51	2.14
	*TIR1*	g19938_i0	−1.88	0	0	0.00
	*IAA*	g19796_i0	−2.35	0	−3.44	2.11
	*IAA*	g1991_i0	−2.09	0	−3.07	1.60
	*IAA*	g26830_i0	−1.97	0	−2.05	2.03
	*IAA*	g31101_i0	−2.92	0	−5.34	2.29
	*IAA*	g7531_i0	−1.92	0	−2.58	1.83
	*IAA*	g9174_i0	0	0	−2.06	0.00
	*ARF*	g16528_i0	0	0	−1.73	0.00
	*GH3*	g17453_i0	−7.06	2.75	0	0.00
	*GH3*	g17633_i0	0	−2.88	−3.21	0.00
	*GH3*	g47838_i0	0	−7.45	0	0.00
	*GH3*	g52386_i0	−7.46	0	0	6.98
	*SAUR*	g12941_i0	0	5.88	0	0.00
	*SAUR*	g16730_i0	10.09	0	7.32	0.00
	*SAUR*	g27977_i0	−4.75	0	−3.13	4.82
	*SAUR*	g3480_i0	0	0	2.8	0.00
	*SAUR*	g34180_i0	−2.18	0	0	0.00
Plant hormone (ABA)	*PYR/PYL*	g14922_i0	−2.66	−2	−3.05	1.97
	*PYR/PYL*	g17896_i0	−4.24	0	−7.58	3.64
	*PYR/PYL*	g18698_i0	−2.72	0	−2.5	1.44
	*PYR/PYL*	g19461_i0	−2.87	0	−3.97	2.36
	*PYR/PYL*	g39537_i0	−2.32	0	−1.72	2.24
	*PP2C*	g12123_i0	0	3.2	0	−2.44
	*PP2C*	g2605_i0	2.05	0	1.62	−1.64
	*PP2C*	g3824_i0	3.75	0	4.08	0.00
	*PP2C*	g5529_i0	2.76	0	2.72	0.00
	*PP2C*	g8795_i0	2.26	0	2.9	−1.53
	*SNRK2*	g14505_i0	0	0	1.36	0.00
	*SNRK2*	g66651_i0	1.31	0	1.18	0.00
	*SNRK2*	g700 5_i0	2.78	0	4.22	−1.63
	*SNRK2*	g77303_i0	2.72	0	4.26	0.00
	*SNRK2*	g8864_i0	0	0	0	8.56
	*ABF*	g25840_i0	1.59	0	1.35	0.00
	*ABF*	g32494_i0	−1.76	0	0	1.38

Further, we searched for DEGs related to fatty acid biosynthesis since lipid content increased after half-waterlogging treatment in Adiantum leaves. In case of DW05, two Acetyl-coenzyme A carboxylase carboxyl transferase subunit alpha (ACCase) transcripts were differentially expressed. On the other hand, seven of 11 long chain acyl-CoA synthetase 9 (ASCL) transcripts were upregulated in DW10. This is consistent with the increased lipid biosynthesis in DW10 compared to CK. A similar trend was noted for the genes enriched in fatty acid elongation pathway; six transcripts annotated as 3-ketoacyl-CoA synthase 4 (KCS) were differentially regulated in DW10 and no transcript of KCS was differentially regulated in DW05. Similarly, a cytochrome P450 86B1 (CYP86B1, *g12531_i0*) and peroxygenase 2 (PXG2, *g235_i0*) enriched in cutin, suberine, and wax biosynthesis were only upregulated in DW10 compared to CK, whereas none was expressed in DW05. These expression patterns are consistent with the metabolome profile changes that half-waterlogging increased the fatty acid contents (Supplementary Table S5). These changes suggest that half-waterlogging for 5 days triggers changes in a small number of genes related to fatty acids compared to the stress for 10 days.

For the alpha-linoleic acid metabolism, we observed that triacylglycerol lipase (TGL; *g5204_i0*) was downregulated in DW05 and DW10, whereas linoleic acid content increased after half-waterlogging stress. The LOX2S (*g3327_i0*) and OPR (*g25288_i0, g8118_i0*) were upregulated in both DW05 and DW10. However, AOS, AOC, DOX1, LOX2S, MFP2, and ACOX1 were upregulated, and LOX2S, OPCL1, OPR, AOS, and LOX2S downregulated in DW10 only. For linoleic acid metabolism, only transcripts were differentially regulated for DW05 (*TGL4; g5204_i0,* downregulated and LOX2S; *g3327_i0*, upregulated). These two transcripts followed a similar pattern in DW10. Three additional transcripts for LOX2S and two for LOX1 were differentially regulated in CKvsDW10. These expression changes indicate that half-waterlogging causes significant changes in linoleic acid and alpha-linoleic acid metabolism.

Finally, we checked for the expression changes in phenylpropanoid biosynthesis and flavonoid biosynthesis pathway genes. Only 10 transcripts enriched in phenylpropanoid biosynthesis were differentially regulated in CKvsDW05 compared to 46 transcripts in CKvsDW10. The HCT (*g24768_i0, g14561_i0*) were upregulated for DW05 and DW10. For DW05, in addition to HCT*,* only phenylalanine ammonia lyase (PAL; *g31530_i0*), 4-coumarate--CoA ligase (4CL; *g10471_i0*), caffeic acid 3-O-methyltransferase (COMT; *g6175_i0*), cinnamyl alcohol dehydrogenase (CAD; *g25451_i0*), cationic peroxidase (PNPC; *g33460_i0, g29160_i0*) were differentially expressed. Of these only, a 4CL, CAD, and a POD were downregulated in DW05, whereas all other were upregulated. On the contrary, large scale transcriptomic changes happened in DW10 leaves. In addition to above mentioned HCT, PAL, 4CL, COMT, CAD*,* and PNPC transcripts, following transcripts were also differentially expressed; cinnamic acid 4-hydroxylase (CA4H), caffeoyl-CoA O-methyltransferase (CCoAMT), 1-Cys peroxiredoxin (PRDX6)*.* As for the flavonoid biosynthesis, only five transcripts were upregulated in DW05 and the same were also upregulated in DW10. These transcripts included flavonoid 3′,5′-hydroxylase (*F3′5′H; g14562_i0*) and HCT (*g24768_i0, g14561_i0, g12450_i0*)*.* In addition to these, a higher number of genes were differentially expressed in DW10 including HCT, CA4H, CCoAMT*,* and CHS in DW10. These expression changes clearly indicate their roles in higher flavonoid biosynthesis in half-waterlogging treated Adiantum leaves. Also, relatively higher FPKM values of these genes as well as expression of a higher number of transcripts in DW10 compared to CK and DW05 indicate that prolonged half-waterlogging causes a significant increase in flavonoid biosynthesis.

#### 3.3.2 Transcriptomic response confirms metabolomic changes in Adiantum leaves in response to drought stress

**FIGURE 6:**
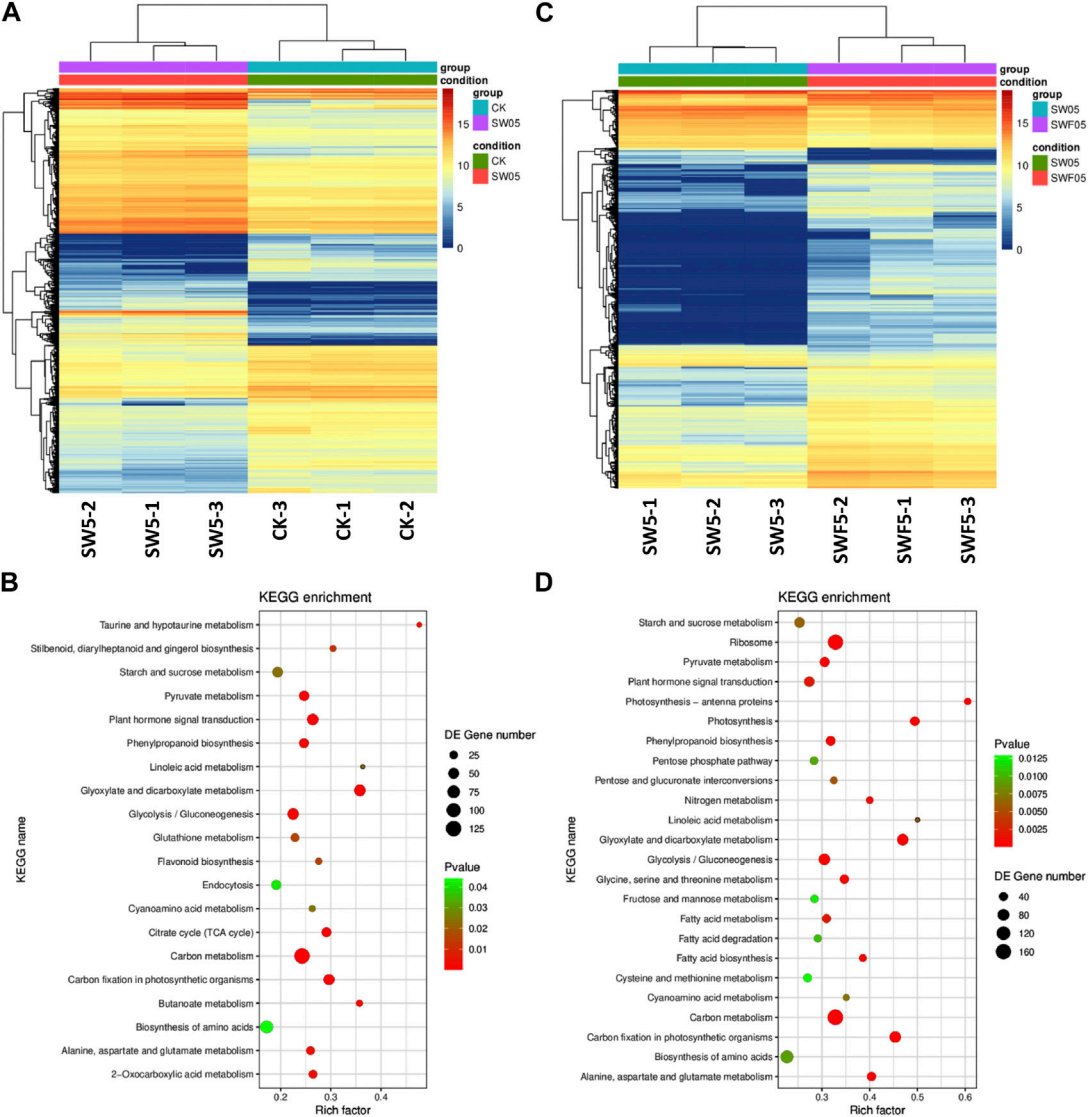
Drought stress and rewatering after drought stress driven transcriptome changes in Adiantum leaves. **(A)** Heat map representation of expression profiles for differentially regulated genes in response to 5 days of drought stress, **(B)** Heat map representation of expression profiles for differentially regulated genes after 5 days of rewatering. **(C)** Scatter plot showing the pathways to which the DEGs were significantly enriched upon drought stress and, **(D)** Scatter plot showing the pathways to which the DEGs were significantly enriched after 5 days of rewatering. Where, CK = control, SW05 = drought stress for 5 days, and SWF05 = 5 days after rewatering.

As we observed a significant increase in phenolic acids, nucleotides and derivatives, alkaloids, and flavonoids in Adiantum leaves after drought stress, we looked for the changes in the expression of related pathway genes. Of the 5,602 DEGs, 2,833 and 2,769 were up- and downregulated in SW05 compared to CK, respectively (Figure 4A; Figure 6A). These DEGs were significantly enriched in biosynthesis of secondary metabolites, flavonoid biosynthesis, starch and sucrose metabolism, linoleic acid metabolism, carbon metabolism, glycolysis/gluconeogenesis, and plant hormone signal transduction pathways (Figure 6B). Overall, the KEGG pathway enrichment results for transcriptome are consistent with the metabolome results.

Since the metabolome profiles showed changes in sugar-related pathway metabolites, e.g., those enriched in pentose and glucuronate interconversions, fructose and mannose metabolism, galactose metabolism, and starch and sucrose metabolism, therefore, we observed changes in expression of related genes and pathways. A total of 56 transcripts enriched in glycolysis pathway were differentially expressed. Mainly, we observed that the aldehyde dehydrogenase (AtALDH4; *g44078_i0*), lactate dehydrogenase (LDH; *g7669_i0*)*,* pyruvate decarboxylase (PDC; *g6937_i0*)*,* glyceraldehyde-3-phosphate dehydrogenase (GAPDH; *g33911_i0* & *g29523_i0*)*,* Pyruvate dehydrogenase (PDHA; *g17423_i0*) were highly upregulated in response to drought. On the other hand, phosphoenolpyruvate carboxykinase (PEPCK; *g41641_i0, g37686_i0*), alcohol dehydrogenase (ALDH; *g17467_i0, g19494_i0, g35797_i0*)*,* lactate dehydrogenase (LDH; *g37686_i0*) were highly downregulated in SW05 compared to CK. These changes are consistent with the observed increased accumulation of sugars in SW05 compared to CK.

As multiple pathways converge on TCA cycle, thus we looked for the upregulated genes and found that out of 54 DEGs, the malate dehydrogenase (MDH; *g31208_i0*), pyruvate dehydrogenase E1 (PDHE1-A; *g17423_i0, g21782_i0*), and citrate synthase (CS; *g17995_i0, g15460_i0*) were highly upregulated in SW05 compared to CK. Interestingly, we found that seven of the eight transcripts enriched in fatty acid biosynthesis pathway that were enriched as long chain acyl-CoA synthetase 9 (ACSL) were upregulated in SW05 compared to CK. Additionally, the genes associated with the fatty acid elongation, i.e., 3- ketoacyl-CoA synthase or KCS were upregulated (*g5707_i0,* & *g20796_i0*) in SW05 compared to CK. These changes confirm the metabolome profiles of the Adiantum leaves where lipid accumulation increased after drought. This was also confirmed by the upregulation of the peroxygenase (*g235_i0*) related to cutin, suberine, and wax biosynthesis (ko00073) pathway in response to drought stress.

We investigated changes in related genes to understand the downstream effects of alterations in the photosynthesis pathway, which is essential for plant survival and development. Drought significantly affected photosynthesis as evident by the downregulation of 18/19 transcripts. The only upregulated gene was photosystem II CP43 chlorophyll apoprotein (PSII43; *g495_i0*). Whereas, ATP synthase (atpF), plastocyanin (petE), ferredoxin NADP reductase (petH), oxygen evolving enhancer protein (PsbO), PSII subunit P-1 (psbP), photosystem II reaction center W protein (psbW), and photosystem II 10 kDa polypeptide (psbR) were all downregulated. These indicate that drought significantly affected photosynthesis in Adiantum. This affected the downstream pathways such as carbon fixation, where 33 of 48 transcripts showed reduced expressions after drought stress.

Two metabolites associated with linoleic acid metabolism were up-accumulated in SW05 compared to CK. These metabolites are produced after the linoleate is broken down. In this regard, we observed that all transcripts annotated as lipoxygenase (LOX, *g2769_i0, g39732_i0, g534_i0, g6571_i0, g836_i0, g3938_i0*) and others (*g39732_i0, g3033_i0, g16198_i0, g37063_i0, g20434_i0, g22002_i0, g5204_i0, g18043_i0, g3938_i0*) were downregulated.

As discussed above (section 3.2.2), in response to drought stress, more than 70% of the DAMs belonged to lipids, phenolic acids, terpenoids, and flavonoids. We checked for the changes in expression of related genes. Only three DEGs annotated as geranylgeranyl diphosphate synthase (GGPS, *g9415_i0*)*,* HMG-CoA reductase (HMGCR, *g8606_i0*), and dehydrodolichyl diphosphate synthase (*DHDDS, g14312_i0*) showed increased expressions in SW05 compared to CK. However, other genes involved in terpenoid biosynthesis were not differentially expressed or screened out based on the selection criteria. Therefore, it is possible that the reduced terpenoid content in SW05 could be due to regulation of some other related genes.

Moving further, since the flavonoid biosynthesis increased in response to drought stress, we checked for the changes in the expressions of related genes. Twenty-five transcripts enriched in phenylpropanoid biosynthesis pathway were differentially regulated. These include phenylalanine ammonia-lyase (PTAL; *g14807_i0*, upregulated), trans-cinnamate 4-monooxygenase (CA4H; *g21065_i0*, upregulated), 4-coumarate-CoA ligase (4CL, *g362_i0,* upregulated), and hydroxycinnamoyl-coenzyme A quinate transferase (HCT, *g23429_i0, g704_i0, g14561_i0, g20519_i0, g26680_i0, g24768_i0,* upregulated & *g16541_i0, g5363_i0, g18055_i0, g4757_i0, g21913_i0*, downregulated). Concomitantly, 16 transcripts enriched in flavonoid biosynthesis were differentially regulated. These included cinnamic acid 4-hydroxylase (CA4H, *g19622_i0,* downregulated), chalcone synthase (CHS; *g39825, g16571,* downregulated), hydroxycinnamoyltransferase (HCT; *g16541_i0, g21913_i0*, downregulated and *g1849_i0, g23429_i0, g704_i0, g33096_i0, g21065_i0, g14561_i0, g14562_i0, g20519_i0, g26680_i0,* upregulated), and flavonoid 3′,5′-hydroxylase (F3′5′H, g14562_i0, upregulated). From these observations, it is clear that the upregulation of the PTAL, CYP3A, 4CL, and HCT transcripts is important and suggests drought affected their expressions, which ultimately lead to increased flavonoid biosynthesis (Table 1).

#### 3.3.3 Transcriptome sequencing confirms metabolomic changes in Adiantum leaves in response to rewatering

As evident from the metabolome profiles and transcriptome sequencing results that flavonoids, lipids, nucleotide and derivatives, alkaloids, organic acids, and phenolic acid content are differentially regulated in response to rewatering after drought. We searched for the genes and pathways whose expression changed in Adiantum leaves experiencing drought and then were rewatered after 5 days (Figure 6C; Table 1). For this we compared transcriptome profiles of SW05 with SWF05 and found that 5,200 genes were differentially expressed; 1,087 of which were downregulated in SWF05 compared to SW05, whereas 4,113 were upregulated in the former compared to the later. These DEGs were enriched in 116 different KEGG pathways. Notably, the DEGs were significantly enriched in ribosome, photosynthesis, antenna proteins, glyoxylate and dicarboxylate metabolism, nitrogen metabolism, carbon metabolism, fatty acid metabolism, carbon fixation in photosynthetic organisms, and fatty acid, phenylpropanoid biosynthesis, and plant hormone signal transduction (Figure 6D). We also checked for changes in the glycolysis/gluconeogenesis pathway genes and found that L-lactate dehydrogenase (LDH; *g41641_i0*) was upregulated while, PDHA, PDHB, and pfkA transcripts were downregulated. Other genes such as ALDH, ADH, GAPDH, pckA, and ALDO showed variable expression patterns. These expressions suggest that the drought-stressed/rewatered Adiantum leaves undergo large scale expression changes in the glycolysis related genes and resultantly, the sugar contents are being adjusted in response to improved water supply and resulting photosynthesis.

Interestingly, we observed that almost all the DEGs enriched in photosynthesis were upregulated in response to rewatering in drought-stressed Adiantum leaves. This clearly indicates that rewatering initiates expression changes, which lead to improved photosynthesis in Adiantum leaves. A similar expression trend was noticed for all the DEGs enriched in photosynthesis–antenna proteins pathway and 48/61 DEGs enriched in carbon fixation in photosynthetic organisms. Likewise, nine of 24 genes were differentially regulated (six up and three downregulated) in flavonoid biosynthesis pathway. Interestingly, all the upregulated genes were downregulated/non-differential in CKvsSW05, CKvsDW05, and CKvsDW10. The upregulated genes included HCT (*g16541_i0; g17826_i0; g21913_i0; g5363_i0; g18055_i0*) and CHS (*g19622_i0*) transcripts, although the other HCT transcripts were downregulated/non-differential. These expression changes, particularly, the upregulation of CHS and HCT indicate that flavonoid biosynthesis is affected after rewatering compared to the drought stressed Adiantum leaves. A similar trend was observed for the phenylpropanoid biosynthesis related genes.

Among the DEGs enriched in linoleic acid pathway 7/10 genes were upregulated in SWF05 compared to SW05 as well as DW05 and DW10, indicating that rewatering caused readjustments in the linoleic acid biosynthesis pathway. Whereas, in case of alpha-linoleic acid metabolism, most of the genes were increasingly expressed in SWF05 compared to both SW05, DWO5 as well as DW10.

Interestingly, in SW05vsSWF05, a contrasting expression profile of the major genes enriched in fatty acid biosynthesis as well as fatty acid elongation pathways showed. A similar pattern was observed for the genes expressed in DW05/DW10. In other words, if a gene/transcript is downregulated in drought or half-waterlogging conditions, this gene is upregulated in rewatering conditions and *vice versa* (Table 1; Supplementary Table S5). Particularly, we observed downregulation ACSL and accA/B, KCS, ADH1 transcripts in SWF05 compared to SW05. However, other transcripts of ALDH, ACOX, FASN, and fabG/H were also upregulated, indicating that specific transcripts were downregulated in response to rewatering and the changes in lipids (fatty acids) were variable at the time of the sampling. Overall, these expression trends suggest that rewatering after drought stress in Adiantum leaves leads to changes in the fatty acid profiles.

#### 3.3.4 Expression changes in phytohormone signaling genes in response to drought, rewatering, and half-waterlogging stresses

Since we observed increased accumulation of abscisic acid in DW05 as well as DW10 compared to CK, therefore, we looked for the changes in the expression of ABA signaling genes. All PYR/PYL transcripts showed reduced expression in DW05 compared to CK, while only one PP2C was upregulated in the former compared to CK. These expression changes are not consistent with the changes in the ABA content. Similarly, we observed that all PYR/PYL transcripts were downregulated in response to half-waterlogging stress. Whereas, the PP2Cs showed upregulation in response to DW10 compared to CK. A similar expression trend was noted for SnRK2 transcripts in DW10, i.e., upregulated compared to CK. One ABF was upregulated in DW10 compared to CK. These changes are not consistent with the increased ABA contents, suggesting the presence of ABA-independent pathway for downstream gene activation, e.g., stomata closure, etc. The rewatering treatment showed lower ABA content compared to DW05 as well as DW10. In this regard, we noted that PYR/PYL transcripts showed upregulation while PP2C, and SnRK2 transcripts were downregulated in SWF05 compared to DW05 and DW10. Taken together, we did not find strong consistency in ABA content changes and ABA signaling related genes in Adiantum leaves under drought stress and rewatering conditions. Finally, the half-waterlogging conditions did not show any changes in ABA content and similarly no expression changes in ABA signaling related genes (Table 1).

Though we did not detect changes in the concentrations of other phytohormones in response to drought and half waterlogging stress or rewatering conditions, we observed changes in the expression of genes related to their signaling pathways. The DW10 significantly affected the expressions of auxin signaling related genes, i.e., IAA, TIR1, GH3, and SUAR. All these genes were downregulated except two SAUR in DW10 as well as SW05 compared to CK, indicating auxin signaling is active both in half-waterlogging as well as drought conditions. Whereas, rewatering caused an increase in the FPKM values of most of these genes, implying that the drought driven expression changes are reversed when watered after 05 days. Likewise, the DEGs related to salicylic acid signaling, i.e., PR1 and TGA were downregulated in response to DW10, whereas their FPKM values increased in the rewatered Adiantum leaves. These expression changes indicate that both the auxins and SA are somehow producing responses in Adiantum leaves as a result of half-waterlogging (if prolonged till 10 days) and rewatering (Supplementary Table S5).

#### 3.3.5 Expression changes in ROS scavenging related genes in response to drought, rewatering, and half-waterlogging stress

We also checked the expression changes in ROS scavenging related genes including superoxide dismutase (SOD), catalase (CAT), ascorbate peroxidases (APX), dehydroascorbate reductase (DHAR), monodehydroascorbate reductase (MDHAR), and glutathione reductase (GR). The half-waterlogging for 5 days caused an increase in expression of only one SOD (*g50250_i0*), whereas a large number of SOD, CAT, a DHAR, and two GR were increasingly expressed in DW10 compared to CK. These changes indicate that half-waterlogging for 5 days did not initiate large scale ROS scavenging/homeostasis, whereas when half-waterlogging is prolonged to 10 days, a higher number of ROS were possibly produced as evident from increased expression of most of ROS scavenging genes. Interestingly, rewatering caused reduced FPKM values of most of these genes indicating that it relieved the drought stress effects in the Adiantum leaves. Similar to DW10, the drought stress also caused increased expressions of SOD, CAT, APX, DHAR, and GR compared to CK. Thus, these expression changes indicate that drought stress for 05 days and half-waterlogging for 10 days are damaging to Adiantum plants and rewatering can help reduce these effects.

## 4 Discussion

Recent forecasts have shown that the drought as well as precipitation are likely to increase in China (Ma et al., 2021). These forecasts are consistent with the rising global temperatures as well as future forecasts (Munaweera et al., 2022). Since Adiantum is a threatened species in China, grows on a narrow distribution area, and often experiences water stress conditions, it is important to explore the global metabolome and transcriptome changes in response to drought and half-waterlogging stress (Kang et al., 2008). Such data is scarce and its availability will open new research possibilities such as breeding Adiantum with better drought/half-waterlogging tolerance so that it can survive in relatively larger natural distribution areas. Therefore, our data provide preliminary but important information in this regard. Discussed below are the changes in metabolome and respective transcriptome changes in Adiantum leaves in response to drought and half-waterlogging stress as well as under rewatering conditions after drought stress.

### 4.1 Half-waterlogging stress caused a significant increase in primary and secondary metabolites by inducing expression changes in respective pathway genes

Excess water stress induces a series of physiological responses at cellular, molecular, and metabolic levels. Half-waterlogging significantly affects plant growth and development leading to drastic reductions in crop yields (Striker, 2012). Earlier studies have shown that half-waterlogging significantly alters plant-water relationships and the rate of photosynthesis, which leads towards changes in stomata opening/closing, leaf dehydration, and reduction in transpiration (Verma et al., 2021). Our results that photosynthesis, antenna proteins, and carbon fixation in photosynthetic organisms related genes showed a reduction in expression in response to both five and 10 days of half-waterlogging indicate that Adiantum undergoes similar physiological responses (Verma et al., 2021) (Figure; Supplementary Table). Different plant species undergo large-scale metabolic reprogramming under half-waterlogging conditions. For example, in wheat, the starch and sugar levels were mostly disturbed during half-waterlogging, whereas most of the amino acids showed increased accumulation (Herzog et al., 2018). In case of Adiantum, we also found that multiple glycolysis/gluconeogenesis, starch and sucrose biosynthesis, and pentose and glucuronate interconversions pathways enriched metabolites/genes showed differential accumulation/expression between CK and DW05 as well as DW10. In addition, the increased accumulation of flavonoids in Adiantum leaves under waterlogging conditions depicts that similar to other plant species, e.g., *Chrysanthemum morifolium* (Wang T. et al., 2019)*,* soybean (Coutinho et al., 2018), and sweet potato (Lin et al., 2006). These increased flavonoid contents are also consistent with the increased expressions of phenylpropanoid and flavonoid biosynthesis related genes (Supplementary table). Similar results have been previously reported in soybean, where waterlogging induced higher expressions of flavonoid biosynthesis genes and subsequent up-accumulation of flavonoids (Lin et al., 2019). Together with flavonoids, ∼81% of the detected DAMs were highly accumulated in leaves of waterlogged Adiantum seedlings (Figure 3). Transcriptome sequencing of different plant species under half-waterlogging stress has shown that this stress induces lipid-remodeling (Xie et al., 2021). Earlier work on Arabidopsis have shown that genes related to lipid metabolism, fatty acid biosynthesis, and other fatty acid metabolism/elongation related pathways showed changes in expression (Xie et al., 2015). Thus, our results conclude that prolonged half-waterlogging for 10 days significantly affects photosynthesis, flavonoid, amino acid, and biosynthesis of other primary and secondary metabolites.

### 4.2 Drought stress caused a significant increase in a broad range of metabolites by inducing changes in expressions of related genes

Physiological adaptive responses in plants include the changes in the accumulation patterns of the primary as well as secondary metabolites in response to drought stress. The primary metabolites such as amino acids, enzymes, and carbohydrates are essential for plant growth, development, and survival, especially under drought stress (Bechtold et al., 2016). The observations that drought stress significantly increased the amino acid as well as nucleotides and their derivatives in Adiantum leaves clearly suggest that this species responds to drought in a universal way and higher amino acid and nucleotide profiles indicate more protein, primary, and secondary metabolite biosynthesis. The increased accumulation of amino acids signifies that Adiantum also adapts this strategy to have better survival under drought stress similar to soybean (Wang et al., 2022), chickpea (Khan et al., 2019), and rice (Auler et al., 2021). The increased accumulation in flavonoids and linoleic acid is quite consistent with our above statements. The higher flavonoid accumulation suggests that Adiantum seedlings try to mitigate drought stress like other plants such as wheat (Ma et al., 2014), pigeon pea (Meng et al., 2021), Arabidopsis (Nakabayashi et al., 2014), and popular (Ahmed et al., 2021). The flavonoids have been implicated as radical scavengers against oxidative and drought stresses in Arabidopsis (Nakabayashi et al., 2014), thus a similar function is present in Adiantum. This increased flavonoid accumulation is consistent with the upregulation of phenylpropanoid and flavonoid biosynthesis related genes such as HCT, 4CL, COMT, F3′5′H, CCOAMT, and CA4H. An earlier study in wheat had shown that the upregulation of these genes is responsible for the higher flavonoid biosynthesis in response to drought stress (Ma et al., 2014). Thus, drought induced expression changes in these genes, which resulted in higher flavonoid accumulation in Adiantum leaves.

Higher lipid (fatty acid) profiles in plant leaves, particularly the formation of a cuticular layer with higher wax and cutin on leaf surfaces, is closely associated with drought stress tolerance (Zhou et al., 2018). Earlier studies have shown that drought stress progressively reduces the lipid (fatty acid) contents, e.g., in Arabidopsis, which is indicative of damage to membranes (Gigon et al., 2004). However, the higher lipid profiles of SW05, clearly indicate that Adiantum adjusts to drought stress by increasing fatty acid biosynthesis. These findings together with the higher expressions of ASCL, KCS, CYP86B1, and PXG2, are consistent with another earlier report in Arabidopsis that fatty acid biosynthesis related genes’ expression increased in order to improve cuticular lipid biosynthesis (Zhao et al., 2021 and references therein). Apart from fatty acids, studies have also shown that linoleic acid and/or alpha-linoleic content increases in drought tolerant wheat genotype after drought stress (Li et al., 2022). Our findings that linoleic acid content increased in drought stress Adiantum leaves are consistent with this report. This increased content can be associated with the increase in the expression of genes including LOX2S, OPR, AOS, AOC, DOX1, DOX2S, MEP2, and ACOX1. Li et al. (2022) also reported that increased linoleic acid was associated with the upregulation of genes enriched in linoleic acid metabolism pathway. As the linoleic acid act as a modulator of cellular membranes in glycerolipids, extracellular barrier molecules such as cutin and suberin, and other bioactive compounds (He and Ding, 2020), therefore, its increased accumulation can be used a marker for drought responses.

Overall, the downregulation of photosynthesis, antenna proteins, and carbon fixation pathway related genes in drought stress indicates that Adiantum plant undergoes changes that effect the process of photosynthesis (Zargar et al., 2017). This, in turn, has been implicated in impaired PSI and PSII (Giardi et al., 1996), which is consistent with our results. Therefore, our results indicate that drought significantly impacts Adiantum plant so that its photosynthetic potential is compromised. Taken together, our results indicate that drought stress induced changes in primary and secondary metabolite biosynthesis in Adiantum leaves and affects its photosynthesis related genes and pathways.

### 4.3 Rewatering causes reversal of drought induced metabolomic and transcriptomic changes in Adiantum leaves

Rewatering to the drought stressed plants often help the plant to recover from the drought effects. However, plants’ reaction to rewatering differs in different species (Morgan, 1984). In our experiment, we observed that all the metabolite compound classes showed increased accumulation after drought stress. However, rewatering caused a significant reduction in the up-accumulated compounds in SWF05 compared to SW05. These results indicate the reversal of drought induced metabolomic changes. Earlier studies in many plant species such as *Populus nigra*, *Lonicera japonica*, *and Phyillyrea angustifolia,* have shown that the plants can recover from drought effects if rewatered within 15 days (Xu et al., 2010 and references therein). Most importantly, the observations that almost all the photosynthesis and antenna proteins related genes showed increased expression in SWF05 compared to SW05 indicate that Adiantum responses are similar to that of other plants, e.g., wheat (Izanloo et al., 2008). The Australian bread wheat cultivars showed a complete recovery of net photosynthetic rate when rewatered after drought stress (Izanloo et al., 2008). The recovery of photosynthesis also affected the glycolysis/gluconeogenesis pathway as evident from large scale expression changes in related genes. Plants often adapt the changes in glycolysis and TCA cycle to manage energy during activation/deactivation of defense responses under drought stress and rewatering conditions (Echevarría-Zomeño et al., 2009).

The reduction in the sum of relative intensities of lipids in SWF05 compared to SW05 as respective downregulation of most of the fatty acid metabolism, and fatty acid biosynthesis related genes clearly indicates that rewatering triggered reduction in lipid accumulation (Zhao et al., 2021). This further suggests that the adjustment of the water cycle in Adiantum can help plant recover from the drought stress affects such as increased fatty acid profiles (Xu et al., 2010). This proposition is based on transcriptome and metabolome profiles and further exploration of the leaf traits will confirm if rewatering resulted in a reduced cuticular lipid, cutin, and wax on the leaves. The reversal of the drought driven changes also included relatively lower total flavonoid contents in SWF05 compared to SW05. This is due to the reduced expression of the major genes enriched in flavonoid biosynthesis pathway. Since the overaccumulation of flavonoids is associated with increased tolerance against oxidative and drought stress (Nakabayashi et al., 2014), therefore, the reduction of these drought stress responsive compounds (Urano et al., 2009) in response to rewatering clearly indicates that rewatering relieves Adiantum from drought stress effects. This flavonoid alteration behavior can be used for manipulating flavonoid biosynthesis genes to change drought sensitivity in Adiantum. Taken together, our combined transcriptome and metabolome data indicate that the drought stress driven changes in Adiantum can be reversed by rewatering the stressed plants.

### 4.4 Phytohormone signaling and ROS scavenging are activated in response to drought and half-waterlogging stress

Apart from flavonoids, the accumulation of organic acids, particularly ABA in leaves of the Adiantum seedlings under drought and half-waterlogging stress is noteworthy as endogenous ABA can induce developmental changes in half-waterlogged plant tissues (Chen et al., 2010). Other studies have shown that ABA synergistically act with GA and ethylene and confer plant adaptations to flooding/submerged conditions (Steffens and Sauter, 2005). Our findings that DEGs related to ABA, GA, and other phytohormones were differentially expressed in CKvsDW05, CKvsDW10, SWF05vsSW05 indicate that these hormones play roles in both drought and half-waterlogging stresses. However, a key observation that the ABA signaling related genes’ expressions were not consistent with the changes in its accumulation pattern indicates that in Adiantum, the drought/half-waterlogging stress responses maybe ABA-independent. Similar claims have been made earlier for the lycophytes and ferns (Brodribb and McAdam, 2011). Nevertheless, the increased accumulation of ABA in these stresses should be further explored.

Reactive oxygen species play essential roles in plant growth and development, and to maintain normal plant growth. Under stress conditions, ROS levels are increased, which significantly damages plant cells. Thus, plants activate the ROS scavenging mechanisms (Huang et al., 2019). The observation that the expressions of CAT, SOD, POD, APX, DHAR, and GR were increased in response to drought and half-waterlogging stress indicates that Adiantum leaves experience higher ROS generation and their scavenging mechanisms are activated under these stresses. Similar mechanisms are present in a diverse range of plant species (Steffens et al., 2013; Verma et al., 2019). After the drought stress, plants are rewatered, and the levels of the ROS decrease in the recovered plants such as in Kentucky bluegrass (Bian and Jiang, 2009). Such a decrease could be followed by noticing a reduction in the expressions of the ROS scavenger genes/enzymes (Xu et al., 2011). Our results that most of these genes showed reduced FPKM values in rewatered Adiantum leaves compared to the drought-stressed ones clearly indicate that ROS homeostasis is activated after rewatering the drought-stressed Adiantum seedlings. Taken together, our combined metabolome and transcriptome profiling of Adiantum leaves indicate that drought and half-waterlogging stresses activate plant-hormone signaling as well as ROS scavenging pathways. The changes in these pathways are reversed when the drought-stressed seedlings are rewatered.

## 5 Conclusion

In this study, we observed the metabolome profiles and transcriptome signatures of Adiantum leaves under drought and half-waterlogging stress and rewatering conditions. We conclude that the Adiantum seedlings respond to drought and half-waterlogging stress by activating multiple pathways related to biosynthesis of primary and secondary metabolites. The major primary and secondary metabolites produced under these stresses in Adiantum leaves include amino acids and derivatives, nucleotides and derivatives, flavonoids, terpenoids, alkaloids, and phenolic acids. The contents of the metabolites (except terpenoids) increase under these stresses and these changes are associated with the expression changes in respective pathway genes. The rewatering has a recovery impact on the Adiantum seedlings as evident from the reversal of the metabolome changes and expression patterns. These responses are accompanied by the activation of phytohormone signaling and ROS scavenging pathways.

## Data Availability

The datasets presented in this study can be found in online repositories. The names of the repository/repositories and accession number(s) can be found in the article/Supplementary Material.

## Supplementary material

SUPPLEMENTARY FIGURE S1 Group principal component analysis based on metabolome profiles in the stress treated and control Adiantum leaves. Where, CK = control, DW05 = half-waterlogging for 5 days, DW10 = half-waterlogging for 10 days, SW05 = drought for 5 days, and SWF05 = day five after rewatering.
