# Supplementary figures and images for: Metabolomic and transcriptomic responses of Adiantum (Adiantum nelumboides) leaves under drought, half-waterlogging, and rewater conditions
Source: Front Genet. 2023 Apr 17;14:1113470. doi: 10.3389/fgene.2023.1113470 (PMC10149873; doi:10.3389/fgene.2023.1113470)

2D PCA Plot

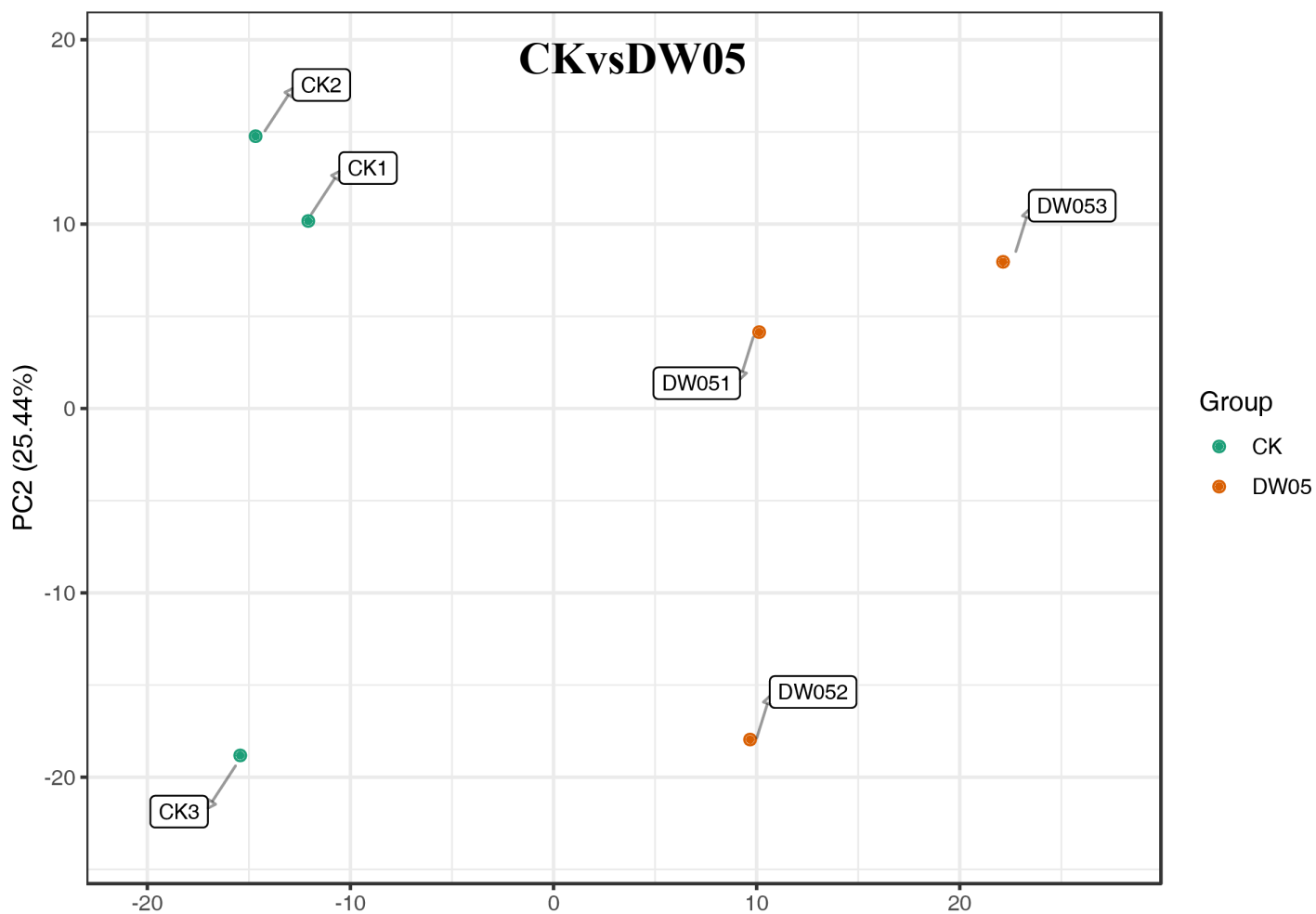

2D PCA Plot

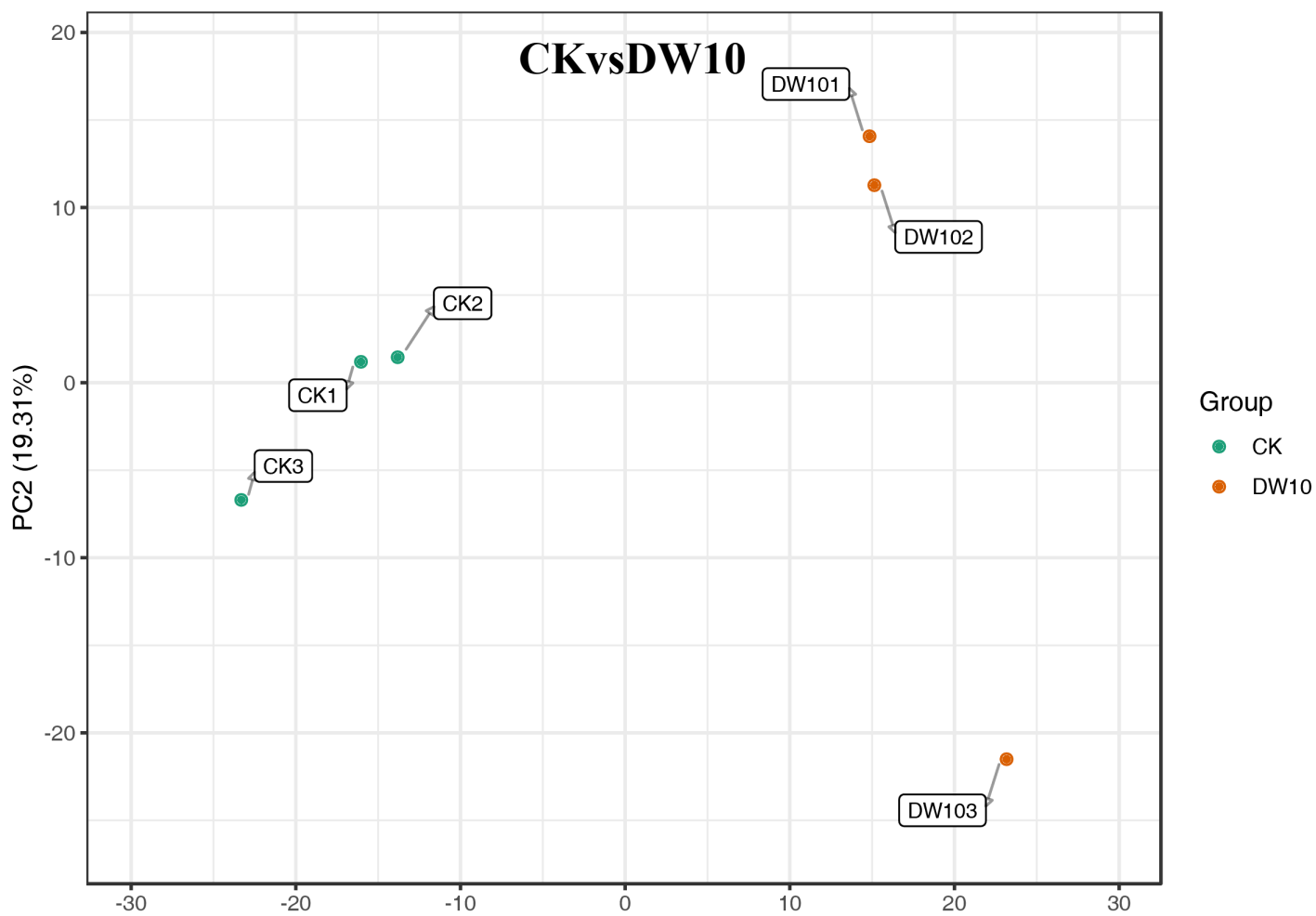

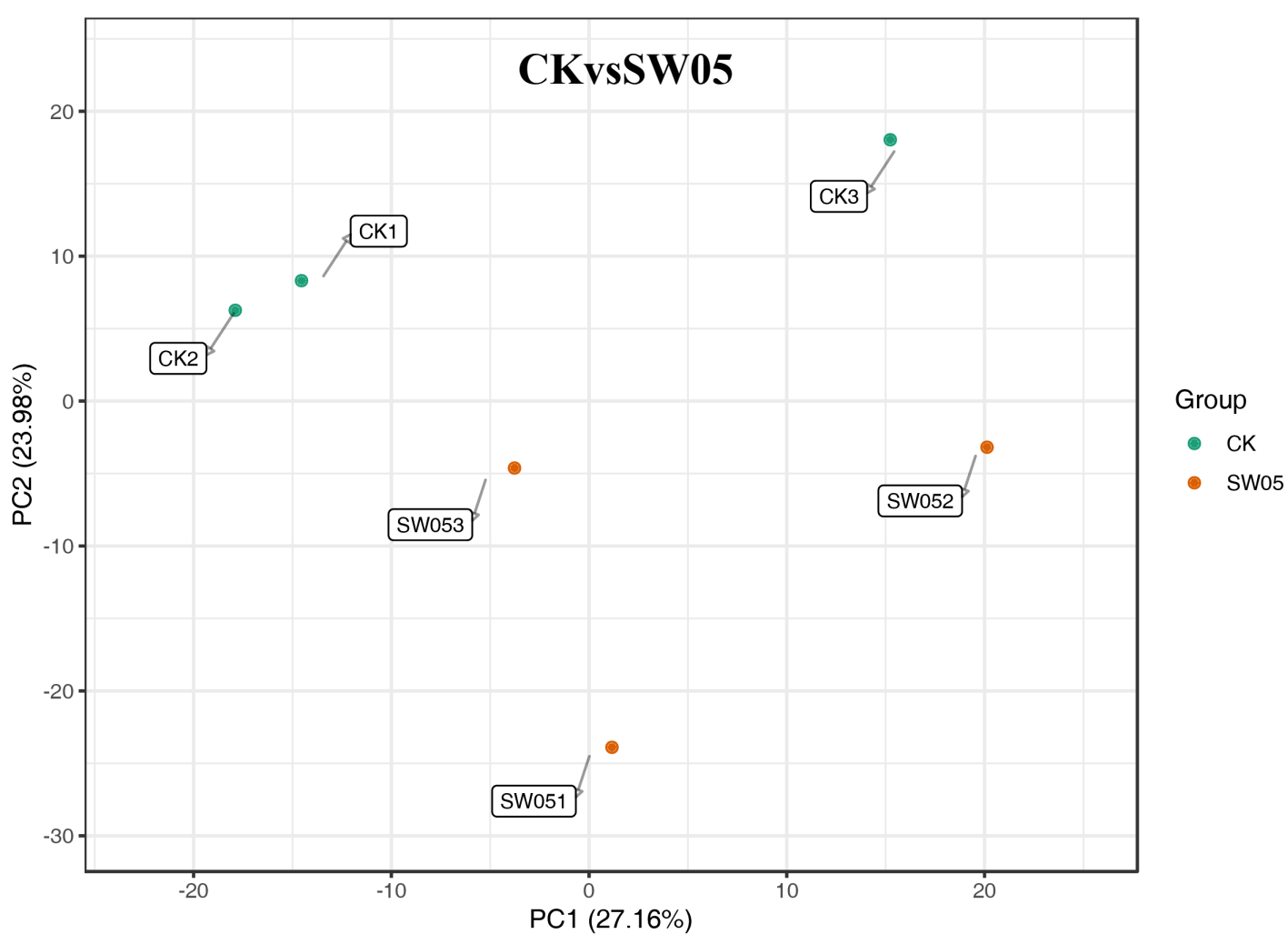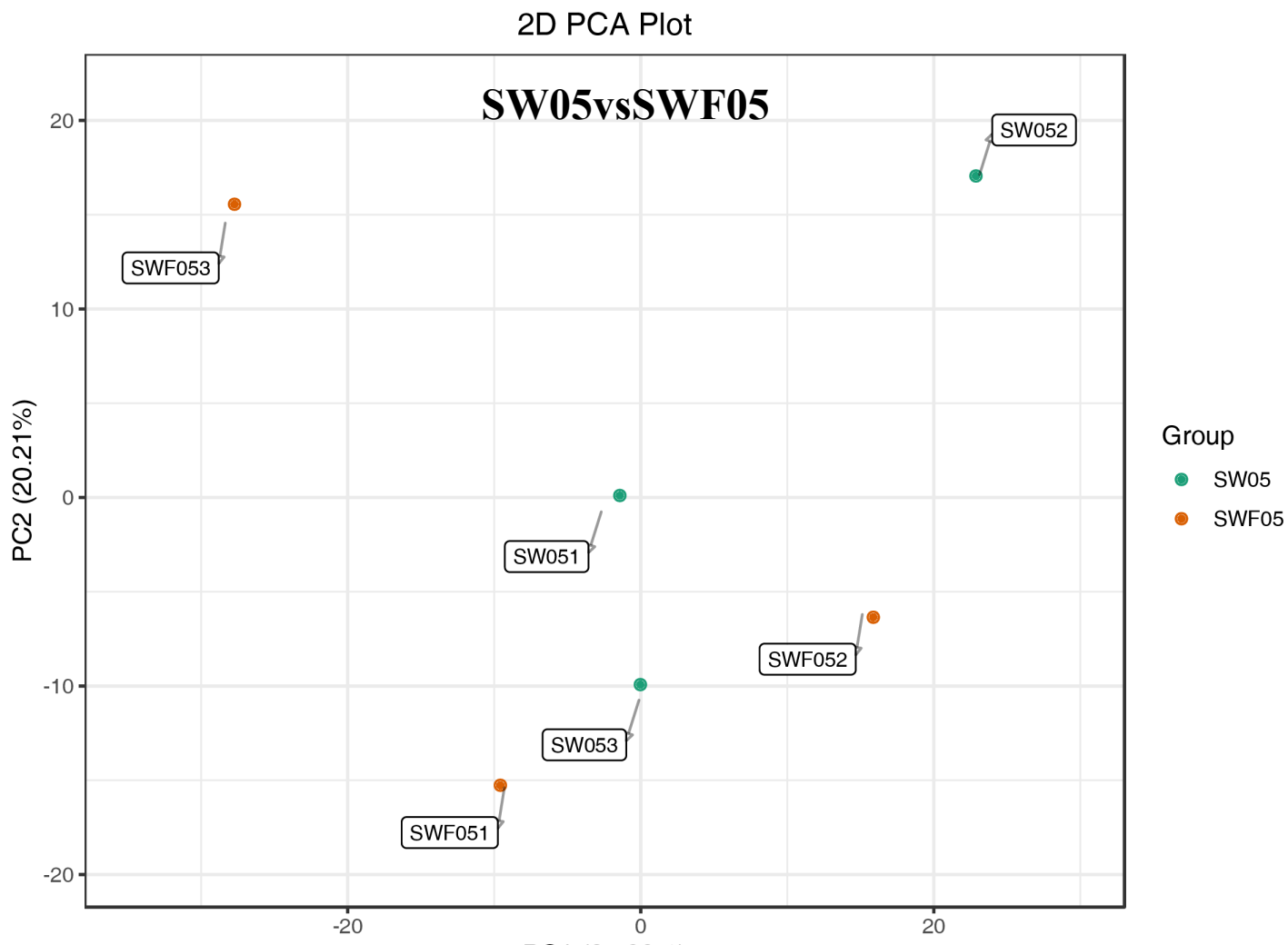

Supplement: Supplementary file 1 [file DataSheet1.PDF]
